# Can Graph Machines Accurately Estimate ^13^C NMR Chemical Shifts of Benzenic Compounds?

**DOI:** 10.3390/molecules29133137

**Published:** 2024-07-01

**Authors:** François Duprat, Jean-Luc Ploix, Gérard Dreyfus

**Affiliations:** Chimie Moléculaire, Macromoléculaire, Matériaux, ESPCI Paris, PSL University, 10 Rue Vauquelin, 75005 Paris, France; jean-luc.ploix@espci.psl.eu (J.-L.P.); gerard.dreyfus@espci.psl.eu (G.D.)

**Keywords:** chemical shift, graph machines (GM), machine learning, structured data, Docker

## Abstract

In the organic laboratory, the ^13^C nuclear magnetic resonance (NMR) spectrum of a newly synthesized compound remains an essential step in elucidating its structure. For the chemist, the interpretation of such a spectrum, which is a set of chemical-shift values, is made easier if he/she has a tool capable of predicting with sufficient accuracy the carbon-shift values from the structure he/she intends to prepare. As there are few open-source methods for accurately estimating this property, we applied our graph-machine approach to build models capable of predicting the chemical shifts of carbons. For this study, we focused on benzene compounds, building an optimized model derived from training a database of 10,577 chemical shifts originating from 2026 structures that contain up to ten types of non-carbon atoms, namely H, O, N, S, P, Si, and halogens. It provides a training root-mean-squared relative error (RMSRE) of 0.5%, i.e., a root-mean-squared error (RMSE) of 0.6 ppm, and a mean absolute error (MAE) of 0.4 ppm for estimating the chemical shifts of the 10k carbons. The predictive capability of the graph-machine model is also compared with that of three commercial packages on a dataset of 171 original benzenic structures (1012 chemical shifts). The graph-machine model proves to be very efficient in predicting chemical shifts, with an RMSE of 0.9 ppm, and compares favorably with the RMSEs of 3.4, 1.8, and 1.9 ppm computed with the ChemDraw v. 23.1.1.3, ACD v. 11.01, and MestReNova v. 15.0.1-35756 packages respectively. Finally, a Docker-based tool is proposed to predict the carbon chemical shifts of benzenic compounds solely from their SMILES codes.

## 1. Introduction

In the field of synthetic organic chemistry, it is imperative for researchers to determine the structure of an unknown or newly prepared compound, or to verify its supposed (and expected) structure. This process, still known as structure elucidation, relies on a battery of spectroscopic analyses, including NMR techniques. Since the very early 1960s, NMR spectroscopy has undoubtedly been one of the main analytical tools applied to the most diverse challenges. As such, it is a powerful enough technique to obtain, from an NMR spectrum recorded for a given structure, information that is relevant, if not sufficient, to fully characterize it. A brief review of the basics of carbon-13 NMR is given in Section S1 of the Supporting Information (hereinafter referred to as SI), with the ^13^C spectrum of 2-methoxytoluene being used as an example (Figure S1).

One of the first methods used to simply estimate the chemical shift of an atom, based on the notion of increments, is also presented in Section S1. It is illustrated for 2-methoxytoluene, whose structure is quite simple since the benzene ring carries only two substituents. To take into account the numerous electronic effects of the substituents and the tedious calculations that may occur in the case of a polysubstituted ring, programs that simulate the manual calculation process based on additivity rules have been published [1,2]. In particular, Hearmon et al. proposed a microcomputer prediction based on previously published ^13^C chemical-shift values for aromatics [3], for computing carbon chemical shifts in substituted benzenes [4]. Only a few prediction examples are given in the paper, but the program seems to be quite efficient and can work with several solvents. Above all, it has the advantage of handling a large number of substituents and producing results quickly. Pretsch et al. report a standard deviation of 5.5 ppm for a first rough test performed by predicting 168,807 ^13^C chemical-shift values [1]. This approach is still implemented in some commercial software [5].

At the same time, authors started proposing Quantum Structure–Property Relationship type (QSPR-type) prediction models to estimate the chemical shifts of carbons in several chemical families using multiple linear regression (MLR) [6,7,8]. The descriptors used to feed these regressions are either topological (e.g., atom and valence counts, connectivity indices); geometrical, which encode information such as throughspace distances to other atoms in the molecule (e.g., the number of heavy atoms contained in a spherical shell at a given distance from the carbon center); or electronic (based on partial charges). To compute those geometric descriptors that are many in most of the models studied, three-dimensional representations of structures are required [9]. Shortly afterward, as computer processing power improved, the performance of these models was enhanced by the use of artificial neural networks [6,7,8,9,10,11,12,13,14]. The main advantage of neural networks over other methods for building empirical models is that they have a greater ability to generalize to new sets of input data, once training has been carried out and optimized [9]. In addition, the results are superior to MLR analysis and additive models, in terms of predictive reliability [15]. This is not surprising because the relationships between ^13^C chemical shifts and molecular structures are essentially non-linear [16]. A criticism of these models is that they are generally specific to a certain class of compounds, which is often of limited size. All these authors use ad hoc descriptors, and their nature often imposes size and structure limits on the molecules of interest [17]. For example, Kvasnička’s models, based on atomic descriptors encoding the substituent structure of a series of monosubstituted benzenes, although giving fairly accurate estimates, were inadequate for predicting the carbon chemical shifts of polysubstituted compounds [12]. Finally, it should be pointed out that, with this type of model, it is necessary to start with a large set of descriptors, obtained after geometric optimization of the molecules, and to select the most relevant ones for the dataset chosen for the study [18].

Meiler et al. have published a single neural network to predict the ^13^C chemical shifts of substituted benzenes [19]. Their model does not use computed descriptors as inputs, but the incremental values of monosubstituted benzenes (the $\delta_{0i}$ term in Equation (S2)), i.e., for a given substituent, four experimental chemical-shift corrections, according to the position of each carbon on the benzene ring relative to this substituent (*ipso*, *ortho*, *meta,* and *para*). In addition, it simultaneously computes chemical-shift values for all six ring carbon atoms, with an optimized neural network having a 24-48-6 architecture. The neural network is trained by a supervised learning method with around 1000 benzene structures containing over 200 different substituents and, then, tested with an independent dataset of some 300 structures. The neural network prediction results are better than those obtained by the incremental prediction methods: the determination coefficients of the scatter plots of predicted shifts versus measured shifts are 0.958 and 0.99 for the incremental and neural network methods, respectively. In addition, the standard errors for the training and test sets are equal to 1.1 ppm for the latter, a significant reduction compared to the 1.5 ppm values computed by the incremental method. The results are impressive, but the number of parameters used by the network—around 1200—raises questions. This is larger than the number of examples presented to the model, entailing the risk of overfitting. It is, therefore, likely that the prediction will not be as accurate for more complex molecules. In addition, the authors note that the average deviation, which is computed as the average of all carbon chemical-shift deviations for molecules with the same degree of substitution, increases with the number of substituents on the benzene ring [19]. Although it is still a vector-machine-type neural network, for which a data vector is presented for each example, it partially encodes the structure of the molecule by mapping for each substituent its relative location on the ring in relation to the carbon for which the chemical shift is calculated. In this respect, it is an ancestor of the graph machines presented in Section 3.1. Unfortunately, such a neural network model cannot be used if the benzene ring is part of a larger polycyclic structure or if the compound carries a substituent that has not been listed [3].

Another approach to ^13^C chemical-shift prediction uses database-based algorithms that search by similarity in a database containing structures for which chemical shifts have been assigned to all carbon atoms. When similar structures are found, the chemical shifts of the atoms of interest can be computed. Prediction quality is highly dependent on the size of the database and the diversity of the structures it contains. The Hierarchical Organization of Spherical Environments (HOSE-code) [20,21] is the most widely used method for predicting chemical displacements [22,23]. It is based on the encoding of an atom-centered structure; a HOSE code encodes the neighborhood information around an NMR-active atom. For each atom in a queried structure, the algorithm describes the atom environment in order to find atoms with a similar environment in a database [18]. The shift value, determined from the retrieved information, is an aggregated chemical-shift value, usually an average or weighted average. An obvious disadvantage of these algorithms is their poor performance for various structures without representative structural fragments in the database. They also work quite slowly, in the order of seconds to tens of seconds for complex structures. Despite these limitations, database-based algorithms have been used in many commercial programs, such as ACD/Labs [24] and NMRPredict [25], or open-source programs, such as Nmrdb [26] and CSearch [27]. They are still often used as a reference to assess the effectiveness of new approaches [28,29,30,31]. On a database of 118 000 individual ^13^C chemical shifts using the ACD/NMR implementation of the HOSE code, the computed mean error is equal to 1.85 ppm, while the standard deviation is equal to 3.05 ppm. The authors note that the approach fails with structures that are underrepresented in the database [32].

In the early 2000s, researchers started using a large number of topological descriptors to describe an atom’s environment in order to predict ^13^C chemical displacements more generally with neural networks. Using a set of 8342 carbon atoms from molecules belonging to a wide variety of families, and containing seven different types of heavy atoms in addition to carbon, namely nitrogen, oxygen, sulfur, and the halogens, le Bret [17] built up a database of chemical shifts with values ranging from −5 to 225 ppm. In his best model, le Bret uses 533 descriptors, most of them topological, to describe the environment of the carbon whose chemical shift is to be estimated, up to its third neighbors. In this particular case, the total number of connections is 9161, and the network learns on 80% of the total dataset. The network yields average prediction errors equal to 2.4 and 4.5 ppm on the training and test (20% of the data) sets, respectively. The fact that the test error is twice as large as the training error suggests overfitting, which, as the author points out in the conclusion, might be ruled out by adding new molecules to the training set. Finally, the author tested his best model on a set of 170 carbon atoms belonging to 34 mono-, di-, or trisubstituted pyridines [33]. The results are probably not what was expected, as the average deviation in prediction for all pyridine carbons is around 7 ppm. The author points out that the dataset contains no substituted heterocyclic aromatic compounds; hence, the significant electron effects caused by the presence of a nitrogen atom in the pyridine aromatic ring cannot be learned by the network.

At the same time, le Bret proposed the first large-scale approach with neural nets, dealing with more than a thousand structures, Meiler et al. reported a PC program that allowed for the computation of the ^13^C NMR spectra of any proposed molecular structure consisting of the covalently bonded elements C, H, N, O, P, S and the halogens using the spherically encoded chemical environments of more than 500,000 carbon atoms [34]. Their program, based on neural nets, enables the prediction of ^13^C NMR spectra, with average chemical-shift deviations of 1.6 ppm at a computation speed around 1000 times faster than the predictions made with the HOSE code. The neural nets have 360 inputs to define five spheres and the additional sum sphere surrounding the carbon atoms, whose chemical shift is to be estimated. Since nine types of atoms are defined, the number of neurons in the hidden layer of the nine networks used for each of these types depends on their frequency in the training dataset. For example, when the first aryl network is trained with 66,433 quaternary aromatic atoms, the chosen number of hidden neurons is equal to 20. For this specific category of aromatic carbons, the root-mean-square deviation (RMSE) is 1.88 ppm for the training set and 1.72 for the test set, which includes 1983 aryl carbons. Similar results are obtained for tertiary (H-bearing) aryl carbons, with RMSE values of 1.57 and 1.81 for the training (113,655 CH) and test (3452 CH) sets, respectively. The averaged figures for the nine networks are 1.97 and 2.10 ppm for the training (510,795) and test (15,716) sets, respectively. Therefore, their work has struck a good balance between accuracy and speed of shift prediction [35]. Shortly afterward, this program was improved by the introduction of an extended hybrid numerical description of the carbon atom environment, resulting in a standard deviation of 2.4 ppm for an independent test dataset of ∼42,500 carbons [36]. As a test of their new method, the authors compared the neural network predictions of the 47 chemical shifts of Taxol carbons with those of a wide variety of other prediction tools. Their networks achieved the second-lowest standard deviation (1.3 ppm). The program, therefore, enables fast and accurate prediction of ^13^C NMR chemical shifts without the need to access databases of molecules or fragments. A similar approach was published by William et al., who built their models from a database of two million ^13^C chemical shifts (207,000 molecules) [18,35]. Additional features have been added, such as a more comprehensive list of atypical atoms and the ability to take solvents into account in the prediction algorithm. Additional flags have also been used to consider the stereochemistry of double bonds. In many cases, cross increments, which refer to pairs of atoms, have been used. In that case, for each pair of atoms separated by no more than two covalent bonds, an independent identifier has been generated and stored (up to three spheres). The authors optimized the main parameters characterizing their models (number of neurons, spheres, cross-increments...), performed trainings using neural networks and partial least squares, and compared the results to those obtained with a database-based implementation of the HOSE code approach. With a test set containing 118,000 ^13^C chemical shifts (11,000 molecules), both methods lead to close RMSEs, equal to 2.45 ppm (NN) and 2.61 ppm (PLS), which are slightly better than the 3.05 ppm obtained with the HOSE approach. It should be noted that the neural network selected has three layers of hidden neurons (100-25-5). The NN model was tested on an external dataset of 92,927 independent ^13^C chemical shifts collected by Robien et al. [37,38] For this collection of shifts, the RMSE for the prediction of the whole set is equal to 3.22. As the trainings are broken down by atom-type sub-databases, RMSEs are also shown for tertiary and quaternary aromatic carbons, which are equal to 1.90 (19,999 CH) and 2.60 (15,289 C), respectively. These results are equivalent to those obtained previously using a comparable approach but applied to smaller test sets (1.81 and 1.72) [34].

Recently, deep-learning (DL) neural networks have made substantial progress in various areas, and a DL-based method for accurately predicting the chemical shifts of both ^1^H and ^13^C nuclei has recently emerged [29,39]. In the second cited paper, convolutional graphical neural networks are applied to learning the ^13^C and ^1^H chemical shifts of 32,538 annotated molecules containing only the elements H, C, O, N, P, S, F, and Cl. For ^13^C shifts, an accuracy of 1.2 ppm mean per-molecule root-mean-squared error (mol RMSE) was obtained on a 177-molecule subset taken from NMRshiftDB, chosen because they had the greatest number of independent spectral measurements. The authors adopt the mol RMSE metric, i.e., the RMSE is first computed for all carbon chemical shifts per molecule, then averaged over all the molecules considered. This result is significantly better than conventional HOSE code-based prediction, whose mol RMSE is equal to 4.24 on the same test set. A new feature, compared with conventional ML methods, which require the process of selecting and creating features from the input data, is that DL allows the creation of the most suitable set of features within the process of training, without any design or involvement by the researcher [40]. These very promising results need to be reproduced on a larger database containing molecules spanning a wider variety of atoms, and taking stereochemistry into account [41]. This technique has been integrated with the NMRshiftDB2 database [42], and Kuhn et al. presented proof-of-concept methods for substructure prediction and compound classification from NMR spectra based on a convolutional neural network [43,44]. In addition, this method can complement any other prediction method, since a confidence interval is computed at the same time for each predicted value. Finally, Kuhn et al. have demonstrated that their deep-learning model is better than more conventional methods (HOSE, SVM) for predicting carbon NMR shifts when the number of spectra used for training exceeds 5000 [45]. Very recently, they have improved their technique by using a type of message-passing graph network block, enabling them to achieve better prediction results using fewer spectra [46].

Finally, a method based on a random forest regression (RFR) algorithm has recently been published for estimating the boron 11 NMR chemical shifts of a series of 1065 BODIPYs and analogs [47]. According to this fragmental approach, the molecular graphs of BODIPYs are broken down into ISIDA (in silico design and data analysis) fragments, whose values, i.e., their number of occurrences in the molecule, are used as RFR inputs. Although this approach gives accurate results for boron, it differs in spirit from ours, since molecular graphs are used to build graph machines without the loss of information. The resulting graph-machine structures are, in fact, isomorphic to the 2D graphs representing the molecules (see Section 3.1). Anyway, while both studies utilize machine-learning techniques, the differences in the target nuclei (^11^B vs. ^13^C) preclude a meaningful comparison with our results.

Alongside these numerous empirical techniques, which are now reaching a form of maturity, there is a second approach to ^13^C NMR chemical-shift prediction, namely ab initio calculations. In principle, ab initio methods can calculate the magnetic properties of any molecular structure, such as shielding tensors, shielding anisotropy, and isotropic chemical displacements with respect to an applied magnetic field and the nuclear magnetic moment. These results can be achieved with high accuracy for entire molecular systems from optimized three-dimensional structures. An important benefit of ab initio methods is that the chemical-shift values obtained are not biased by previous experimental results [36]. But, their most valuable advantage is undoubtedly the handling of compounds bearing exotic fragments or which are freshly synthesized compounds. Ideally, ab initio methods need no adjustment to predict new classes of substances that are either under-represented in current databases, for which spectrum-structure relationships are insufficiently described, or that are not properly handled by any of the empirical or ML-derived methods. Their main disadvantage is that extensive optimization of the spatial structure and/or consideration of multiple conformations, particularly for flexible molecules, makes the calculations required (very) time-consuming and costly. On the other hand, advances in the calculation of NMR properties from first principles made considerable progress with the introduction of Density Functional Theory (DFT) [39,48]. Today’s DFT-based methods can be quite accurate [49] and reasonably turnkey, even if they are time-consuming, and protocols have been developed for their application [50]. Once a mechanism for predicting the error bounds of individual atoms for DFT-based calculations has been developed, this type of prediction could be used in conjunction with the empirical methods. For example, the hybrid functional xOPBE predicts the ^13^C chemical shifts of 38 polycyclic natural products (771 carbon atoms) with very good accuracy, as indicated by the computed root-mean-square deviation of 2.1 ppm [51]. In addition, the development of new types of neural networks that can speed up computation while maintaining excellent prediction is in full swing [51,52,53,54].

To get an idea of the performances of a few methods available today for computing ^13^C chemical shifts for benzene carbons, a set of 22 polysubstituted structures (128 benzenic carbons) published in the chemical literature between 2006 and 2020 was collected. Open-source and commercial software were used to predict the chemical shifts of the ring carbons. The RMSE computed for the 128 chemical-shift predictions with the chosen models are reported in Table 1.

Surprisingly, for this set of 22 fairly simple molecules with a molecular mass of less than 340 Da (see references and detailed results in Table S1 of the SI), the results vary widely depending on the algorithms used. With this set of 22 molecules, the commercial software ACD and MestReNova deliver the best results. Thanks to the ‘ensemble’ technique, when a “predictive” calculation is requested, MestReNova runs several predictors to get the final results. First, the Mestrelab predictor is run—it is actually formed by two different machine-learning predictors trained with different assigned data. Then, the Modgraph predictor, which uses also two different predictors, takes over, and finally, a Bayesian algorithm is triggered to combine all the individual chemical shifts and confidence intervals to obtain the final predicted chemical shifts (and confidence intervals). The ACD/Labs software v. 11.01 uses a dual prediction algorithm, based on neural networks and HOSE code algorithms, but its operation is not known (black-box effect).

The preliminary results reported in Table 1 indicate that it would be highly desirable to have an open-source model capable of predicting benzenic shifts with ppm accuracy, and that is just as efficient as NMRshifDB’s NN method, especially for molecules containing bromine or iodine atoms. Indeed, this family of compounds represents a significant percentage of those for which ^13^C NMR spectra have been published. A SciFinder search on 11 March 2024 [55], for compounds containing an isolated benzene ring, yielded 1,978,095 single-component structures out of a total of 3,083,722 structures for which ^13^C data were available. Of these, 546,838 structures have only one aromatic ring, which is the benzenic ring, and 99% of them (529,755) contain at least one of the eight atoms already mentioned, i.e., oxygen, nitrogen, phosphorus, silicon, or halogen. Furthermore, these compounds have the added advantage, depending on the nature of the ring substituents, of having ^13^C chemical shifts covering over a hundred ppm. 

Indeed, as it is unreasonable to test our approach on too large an ensemble, having a sample of molecules whose chemical shifts can vary by a hundred ppm or so is a good idea. Finally, it should be pointed out that the 540,000 or so benzenic structures described by their carbon NMR spectra represent only a tiny fraction of the total number of molecules that could be obtained by combining the 174 benzenic substituents present in our final database. Considering only tetra-substituted benzenic derivatives with four different substituents, it is, in fact, possible to construct some 1.1 billion different molecules corresponding to 6.6 billion chemical shifts.

Therefore, in the present article, graph-machine modeling (described in Section 3.1) is used to estimate the ^13^C chemical shifts of benzenic carbons measured at 30 °C in a CDCl_3_ solution. Graph-machine models of increasing complexity are designed and trained from a set of 1637 molecules, corresponding to 8431 chemical shifts of benzene-like carbons. A model containing 834 variable parameters is selected after comparing the virtual leave-one-out scores of the trained models. Its ability to generalize is then assessed by predicting the ^13^C chemical shifts of 584 benzenic carbons from a set of 114 fresh molecules. Estimates of carbon chemical shifts that are farthest from the experimental values are analyzed to detect possible errors or insufficient descriptions of molecular structures. A new training dataset of 2026 molecules (10,577 benzenic carbons) is finally built up by merging the previous training and test sets and adding new compounds to extend the range of application of the model while improving its predictive quality. Once trained under the same conditions as before, the model’s performance is compared with that obtained using open-source and commercial software on a new set of 171 molecules combining 1012 benzenic carbons. After validation, the graph-machine model is integrated into a demo software version 1.0 written in Python, which is available for download.

## 2. Results and Discussion

### 2.1. Graph-Machine Model Selection

The selection of the appropriate model, given the available data, was conducted by training the graph machine-based models on the 8431-dataset, according to the methodology defined in Section 3.3, with an increasing number of neurons in the hidden layer of the multi-layer perceptron (MLP) implemented at each node of the graph. In addition to the computation of the virtual leave-one-out (VLOO) score, as defined in Equation (3) of Section 3.3, the root-mean-square training error (*RMSTE*), which is an indicator of the ability of the model to account for the training data, is also computed according to Equation (1):

(1) $$RMSTE=\sqrt{\frac{1}{N_{T}}\sum_{i=1}^{N_{T}}{\left(\delta_{exp.}^{i}-\delta_{est.}^{i}\right)}^{2}}\;,$$

where $N_{T}$ is equal to 8431, $\delta_{exp.}^{i}\;$ is the ^13^C chemical-shift value determined experimentally for carbon *i*, and $\delta_{est.}^{i}$ is the ^13^C chemical-shift value estimated by the model for molecule *i* at the end of the training. The *RMSTE* and VLOO score computations (see Section 3.3) are repeated three times for each number of hidden neurons, so the averages are displayed in Table 2.

As expected, the root-mean-square training error decreases when the model complexity (number of hidden neurons) increases. The variation of the VLOO score follows the same trend as the RMSTE. Increasing the number of hidden neurons from 26 to 30 results in a very small decrease in the average prediction error (0.06 ppm), while the average computation time to estimate the chemical shift for a single carbon atom rises significantly (1.8 s, 50%). Therefore, the graph-machine model with 26 hidden neurons, denoted thereafter by GM26, is kept for subsequent testing. 

### 2.2. Performance of the GM26 Model on the Compounds of the Test Set

In the present section, the ^13^C chemical shifts of 114 compounds, measured with our in-house equipment (see Section 3.2), are estimated by the GM26 graph-machine-based model. For this purpose, estimations of the chemical shift of the 584-carbon test set are computed with the 26 hidden neuron model (selected from Table 2) for three different parameter initializations, using for each sequence the twenty-five models (out of 100) that have the smallest VLOO scores (see SI Section S3 for more details). The means of the resulting three computations are the final predictions for the test set, whose performance, along with that of the training set, is reported in Table 3, and with more details in Tables S3 and S4 of the SI.

The computed root-mean-square errors, respectively, equal 0.5 and 0.7 on the training and test sets (second column, rows 1–2), indicating that the GM26 model performs fine on both sets. As expected, the performances are slightly lower in prediction; however, the RMSE value of 0.7 computed for the test set is even better than the one computed for the training set’s VLOO score (Table 2, antepenultimate column, second row). This demonstrates that (i) the VLOO score on the training set provides an accurate assessment of the generalization ability of the model; (ii) increasing the complexity of the model, given the available data, is not necessary; and (iii) the quality of prediction is very good. The first point (i) is particularly important because, given the size of the training set, a “leave-one-out” (LOO) experiment is difficult to carry out within a reasonable timeframe, with a cycle of 8431 successive trainings having to be carried out in this particular case. The latter point (iii) is also confirmed by the low MAE values equal to 0.4 and 0.5 ppm, respectively, and by the minimum and maximum deviations observed for the GM26 model (Table 3, third column, rows 1–2), which are very moderate. In fact, only seven molecules (nine carbons, 0.1%) in the training set have at least one carbon, whose chemical shift is estimated with an absolute deviation of more than 3 ppm. Meanwhile, four molecules (five carbons 0.8%) in the test set have at least one carbon, whose absolute deviation in shift prediction is greater than 3 ppm.

### 2.3. Scatter Plot of the GM26 Model Estimations on Both Sets

**Figure 1:**
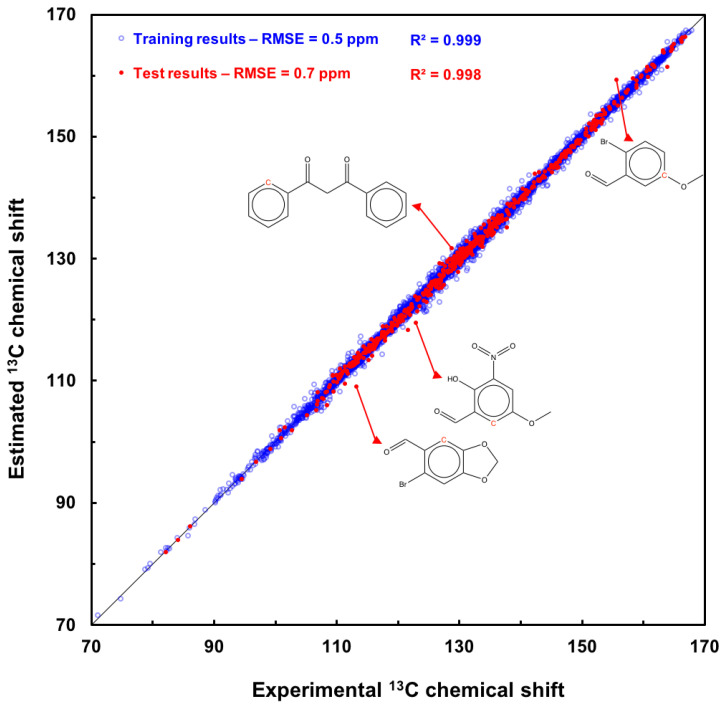
Scatter plot of ^13^C chemical-shift estimations computed by graph machine from SMILES (node function with 26 hidden neurons) for the 1637 compounds of the training set (blue circles) and the 114 compounds of the test set (red filled circles) vs. measured values of the chemical shift. The black line is the bisector of the plot.

To summarize, the ^13^C-δ estimates computed with the GM26 model for the 8341 carbon atoms of the training and the 584 carbon atoms of the test set are plotted against their measured values in Figure 1. The RMSE computed for the two sets is equal to 0.5 and 0.7 ppm, respectively (Table 3), and their determination coefficients R^2^ are above 0.99. Data points (in red) for the four test molecules, which have the largest predicted shift deviation, are also shown in Figure 1. The detailed results are available in Tables S3 and S4 of the SI.

To conclude this section, a computation with the GM26 graph-machine model was performed with the 22-molecule set used to assess the six models in Table 1. The RMSE computed for the 128 predicted shifts, equal to 1.0 ppm, is the best obtained to date, as indicated in Table S1 of the SI. Unlike the commercial models, differentiating between tertiary and quaternary carbons is not necessary for our model; encouraging results are obtained with a reasonable number of adjustable parameters (834). Admittedly, the number of compounds processed is modest, and the scope of the model is limited to benzenic derivatives. But, it does provide accurate predictions of ^13^C chemical shifts for these compounds. 

### 2.4. Analysis of Chemical-Shift Estimates with Large Errors on Both Sets

To understand the limitations of our GM26 model for both datasets, we analyzed the reasons why the chemical-shift estimates for some carbon atoms showed a deviation from the experimental value in excess of 3 ppm. Several factors may explain this discrepancy, including (i) the measured chemical-shift value is incorrect for various reasons, e.g., the sample solvent is dimethylsulfoxide instead of CDCl_3_; (ii) the experimental shift has been wrongly attributed to a given carbon, which most often corresponds to an inversion in the assignment of shifts between two carbon atoms; (iii) the structure used to generate the SMILES does not match the sample form present in the solution; or (iv) the model cannot learn a specific structural feature of the molecule, which is often the case if this feature is poorly represented in the training set [17].

The detailed analysis of the deviations from experimental chemical shifts greater than 3 ppm in absolute value for the shift estimations of nine carbon atoms in the training set and five carbon atoms in the test set is carried out in Section S2 of the SI. The molecules containing the atoms concerned are shown in Table 4, with the corresponding measured and estimated chemical shifts in ppm, the difference between the two, and the case(s) i–iv described above invoked to explain the large discrepancy observed. The last column contains recommendations for improving the estimation of shifts for the listed carbons in a future training database. Based on these recommendations, a new training set is built in Section 2.5, which includes all necessary corrections and the addition of new structures whenever appropriate.

### 2.5. Design of an Extended Graph-Machine-Based Model

Following the in-depth analysis of the results provided by the GM26 model on the two selected sets (Section S2 of the SI), the construction of a larger training set was undertaken in order to (i) minimize the observed deviations, and (ii) extend its prediction domain. Thus, the two previous sets used for model selection and validation were merged to produce a file containing no less than 8983 carbons (1745 molecules), with six molecules having been removed for various reasons. A total of 281 molecules containing new atoms, new functional groups, or highly crowded rings were then added, resulting in a total of 10,577 carbons (2026 molecules). Table 5 summarizes some of the chemical families added, depending on the benzene ring substituent. The complete list of the added molecules, and those removed from the 8431-carbon training set, with justifications, are provided in Tables S6 and S7 of the SI.

A graph-machine model with MLP counting 26 hidden neurons was then built and trained with this new dataset of 10,577 chemical shifts. First, estimations of outlier shifts, listed in Section 2.4, were analyzed for improvement, and then, the performance of the new GM26 model was tested with a small set of 28 benzenic compounds (156 carbon shifts) recently published on SDBS [56] in 2022 and 2023. The results are given in Table 6 and with more details in Tables S8 and S9 of the SI.

Compared with the first GM26 model, the present model is almost as efficient with similar RMSE and MAE for the training set’s carbon shift estimates, as shown by the close values obtained in Table 3 and Table 6 (row 1, columns 2 and 3). In addition, lower values of maximum and minimum deviations are obtained (row 1, columns 5 and 6), and lastly, the RMSE calculated for the shifts incorrectly estimated or predicted with the previous model is lower (row 2, column 2: 2.0 instead of 3.3 for outliers), indicating an improvement in the quality of the estimations. Finally, the prediction of the benzenic carbon shifts of the 28 new molecules, containing all the atom types in the training database except phosphorus and silicon, gives very satisfactory results, with a noteworthy RMSE equal to 1.0 ppm, the limits of which will be discussed in Section 2.7. The very good accuracy (ppm level) obtained with the graph-machine model is of great practical interest to the laboratory chemist. Thanks to our tool, the prediction of chemical shifts of benzene ring carbons can be obtained in a matter of seconds with excellent reliability, enabling, for example, the validity of assignments to be checked on the fly.

### 2.6. Comparison of Known Models with the Graph-Machine-Based Model

To carry out this comparison, a test set containing 171 molecules taken from the literature (1974–2020) and not belonging to the training set was compiled. This selection covers all the types of atoms used in training, each of which is present in at least six different molecules, and all the ^13^C shift values are within our training range. The results of the computations for all models are shown in Table 7.

First, as we do not know which molecules are used to parameterize the models compared to the graph-machine model, the values given in Table 7 are not necessarily significant. Indeed, predictions for molecules belonging to the training set should be removed from the test RMSE computation for the compared models. In the case of the three commercial software packages ChemDraw v. 23.1.1.3, MestReNova MestReNova v. 15.0.1-35756, and ACD v.11.01, the GM model gives better overall chemical-shift predictions for the 171 molecules in the test set, since the computed RMSE equal to 0.9 (row 1, column 1), is the smallest. It can also be seen that the maximum deviations are smaller in the case of the GM model, the largest error in absolute value for all predicted carbon shifts being equal to 3.6 ppm, while it is 27.6, 10.1, and 8.5 ppm for the other methods (columns 4-5). Most importantly, only 0.9% of carbons have a shift predicted by graph machines with an error greater than 3 ppm, compared with 25.3, 10.2, and 9.5%, respectively, for the other methods used (last column). We also report in the last row of Table 7 the prediction results obtained with the NMRshiftDB deep-learning model. For our test set, the shift computation was only possible for 100 molecules (596 atoms), i.e., those containing no bromine, iodine, or silicon atoms. The computed RMSE, equal to 1.1 ppm, is very low, and this good result is confirmed by the small number of carbons (2.5%) that have a predicted shift with an error (in absolute value) greater than 3 ppm. This open-source model is, therefore, very effective for predicting the shifts of benzenic carbons in molecules containing the supported atoms. It is easy to launch a chemical-shift calculation on the dedicated site, even if it can sometimes be a little slow, as pointed out by the authors [57]. It is also worth remembering that this model uses a training set containing almost 10^6^ carbon atoms, i.e., 100 times more than ours. The detail of the resulting ^13^C chemical-shift predictions with the five models for all test molecules is available for download in Table S10 of the SI.

**Figure 2:**
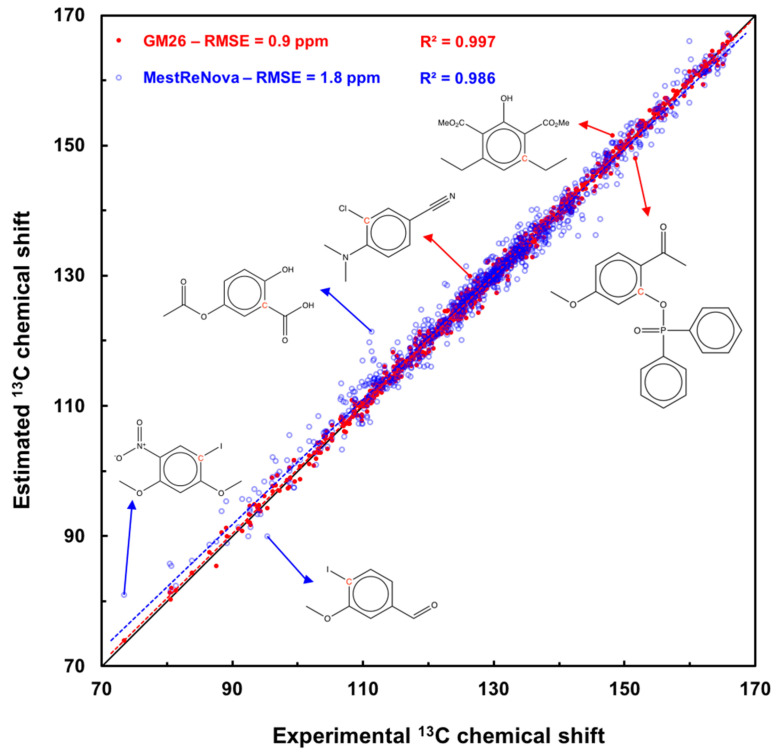
Scatter plot of ^13^C chemical-shift predictions computed by graph machines (red disks) and MestReNova software v. 15.0.1-35756 (blue circles) vs. measured shift values for the 171 molecules of the test set. The black line is the bisector of the plot, and the dashed red and blue lines are the regression lines for the GM and MestReNova plots.

Figure 2 shows a scatter plot of the prediction results with the GM26 and MestReNova models for the 171-molecule test set. The results obtained with the MestReNova software are compared to the graph-machine results because they are the second best, and ^13^C shifts can be predicted for all the carbons in the test set with the most recent version of this software (v. 15.0.1). The fit is very good for the data points (red disks) corresponding to the graph-machine-based model, reflected by the value of the coefficient of determination equal to 0.997, and the red regression line practically coincides with the bisector of the graph in Figure 2. Three molecules, whose indicated red carbons have the largest prediction error, are also shown. The blue points corresponding to predictions obtained with the MestReNova software v. 15.0.1-35756 are further away from the diagonal, indicating less accuracy; this is confirmed by the value of the coefficient of determination equal to 0.986, which is less close to one than that calculated for the GM model. Three molecules whose red carbon shows a shift predicted with a large error are also displayed for information purposes. Note that the graph-machine model correctly predicts the values of these shifts.

### 2.7. Some Limitations of the Graph-Machine-Based Model

**Figure 3:**
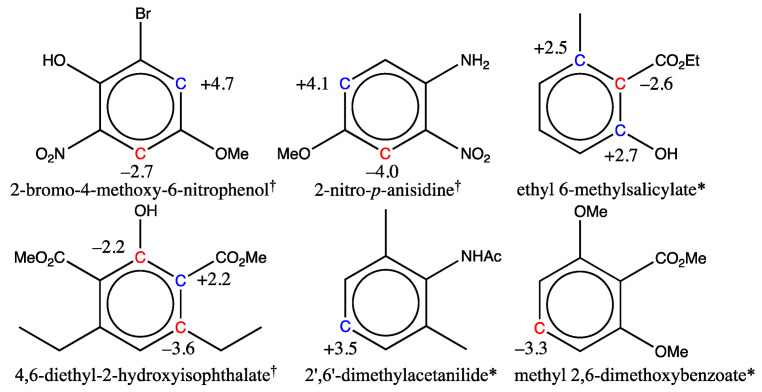
Structure of training * or test ^†^ set molecules with at least one carbon whose shift estimation with the GM26 model shows a large deviation (experimental minus estimated, in ppm). Shifts for blue carbon are underestimated, while they are overestimated for red carbons.

When the final training set was built, six molecules from the first training set were removed because their shifts were not estimated with sufficient accuracy. This choice was necessary for five of them, as the experimental shifts used could not be confirmed by a second reference or were not measured in the appropriate solvent (CDCl_3_). By contrast, for the sixth, 2-nitro-*p*-anisidine, although the numerous references available were mutually consistent, two of its shifts could not be estimated with an accuracy better than 3 ppm. The exact same behavior was observed for the 2-bromo-4-methoxy-6-nitrophenol molecule, which belongs to the test set of 26 compounds used for validation of the new graph-machine model (Section 2.5). The two molecules are shown in the first row of Figure 3 with the deviations observed for the carbon shifts marked in blue (positive deviation from the experimental value) and red (negative deviation). The parallel between the two is obvious, with the same carbons in the alpha position of the methoxy group either having a shift overestimated by the model (red C) or an underestimated one (blue C). The other four molecules all have at least three neighboring carbons bearing substituents that have a certain degree of steric hindrance. The erroneous estimate is then made either on the congested carbons (salicylate and isophthalate) or on the carbon in the *para* position of the main group (acetanilide and benzoate).

So far, we have been unable to explain the discrepancy observed in the prediction of the two carbon shifts for the first two molecules (phenol and anisidine). The sequence of atoms on the benzenic ring that causes these discrepancies is a combination of substituents obtained by starting with nitrobenzene, adding an amino or hydroxy group in the *ortho* position of the nitro group, and positioning a methoxy group in the *para* position to the nitro group. Other molecules in the training set (n°1189 and and n°1545) have such a distribution of substituents and indeed show a similar trend, albeit with smaller deviations (–1.6 and +2.3 ppm, respectively). To confirm that hypothesis, we have predicted the shifts in 4-(methylamino)-2-nitrophenol, which is not part of any set and meets the above criteria. It turns out that, again, significant discrepancies are observed for the shift prediction of the same carbons (−3.9 and +4.6 ppm). For nuclei encumbered by several neighboring substituents, significant deviations in shift predictions are also observed, which is the case for the last molecule of the first row and for all the molecules of the second row in Figure 3. The explanatory effects are not always straightforward for the two salicylates. However, for the last two molecules of the second row in Figure 3, the electronic effects of the sandwiched groups (NHAc and CO_2_Me) are not correctly transmitted in their *para* position, as they are no longer in the plane of the benzenic ring but rather in a perpendicular position. Thus, the amide shielding (−5 ppm) and the ester unshielding (+4 ppm) do not apply in their *para* position, whereas the graph-machine model still takes them into account, resulting in a shift error. We have encountered similar effects in the case of thiophenol and thioanisole derivatives [58,59,60]. Other discrepancies may also arise in the prediction of the shift of highly congested molecules; this is currently a limitation of the model.

**Figure 4:**
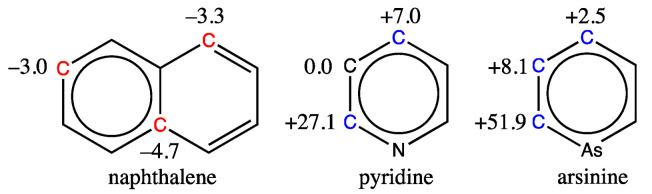
Deviations encountered in the prediction of carbon shifts of three molecules outside the scope of the GM26 model (experimental minus estimated, in ppm). Shifts for blue carbon are underestimated, while they are overestimated for red carbons.

When trying to predict the ^13^C chemical shifts of molecules like naphthalene, pyridine, or arsinine, all shown in Figure 4, the results are far from the mark. That is not surprising, as no fused aromatic bicycles are present in the training database, nor are any heteroaromatics like pyridine or arsinine, the last of which contains an atom that is not part of the training set’s atoms.

Clearly, our model cannot be used to predict the chemical shift of aromatic carbons in molecules very different from those in the training set, but it is still very robust since it computes a shift as long as the provided SMILES code is correct. It is, therefore, essential to make sure that the molecule whose ^13^C shifts are to be predicted is indeed a benzene derivative containing the chemical atoms and functions advertised.

Consequently, we have developed a demonstration tool, based on Docker, fed with the built-in data (chemical shifts of 10577 benzenic carbons, SMILES of molecules). It allows one to replicate the chemical-shift predictions for the 171 compounds on the test set. In addition, version 1.0 of the demo software is also capable of predicting with good accuracy (ppm range) the ^13^C benzenic chemical shifts of any molecule containing carbon, hydrogen, oxygen, nitrogen, halogen, sulfur, silicon, and phosphorus atoms, based on its SMILES code. Details on how to install Docker, download, and use our demo are available in Sections S3 and S4 of the CSdemo-SI.pdf file of the Supporting Information. Readers are then welcome to use the demo software (v. 1.0) to estimate the chemical shift of carbons of the test sets or others that may be of interest. For easy access to the molecules and carbon SMILES used, as well as the references of the papers from which they originate, where applicable, they are given in SI Tables S11–S16.

## 3. Materials and Methods

The design of graph-machine models requires a dataset of measured experimental values, a set of ^13^C chemical-shift values in the present case. An important difference, as compared to the estimation of surface tension, viscosity, or refraction index [61,62,63], is that the property under study is an atomic property instead of a molecular one. Consequently, the carbons of the benzenic molecules must be annotated with their experimental chemical shifts. Numerous experimental chemical-shift databases are either freely available on the Internet or commercially available from various suppliers. In addition to our own collection of ^13^C carbon NMR spectra, we have gathered ^13^C chemical shifts from the well-known spectral database for organic compounds (termed SDBS for spectral database system) provided by the National Institute of Advanced Industrial Science and Technology [56], from the CAS Database [55], or the Landolt-Börnstein collection of carbon-13 NMR data for aromatic compounds [64]. Compared with spectral data extracted from the primary articles referenced in the CAS database, the advantages of using SDBS are as follows. (i) Several thousands of benzenic compounds have ^13^C NMR spectra with chemical-shift values annotated. (ii) In difficult cases, carbon shifts are assigned using several complementary NMR techniques like DEPT, HMQC, or HMBC. (iii) Most of the ^13^C spectra are recorded at 30 °C in deuterochloroform, the most routinely used NMR solvent. (iv) Every compound can be searched with its name, molecular formula, and CAS registry number to access its ^13^C spectrum. An initial dataset of 1637 benzenic compounds corresponding to 8431 measured ^13^C chemical shifts, ranging from 73 to 166 ppm, was compiled from data extracted from the above-mentioned databases. A second dataset was built up from 114 diverse benzenic compounds, for which a ^13^C spectrum was recorded in our laboratory. It contains 584 assigned carbon chemical shifts, with values ranging from 82 to 162 ppm, as well as the SMILES codes corresponding to each carbon (see Section 3.2).

### 3.1. Graph-Machine Modeling

**Figure 5:**
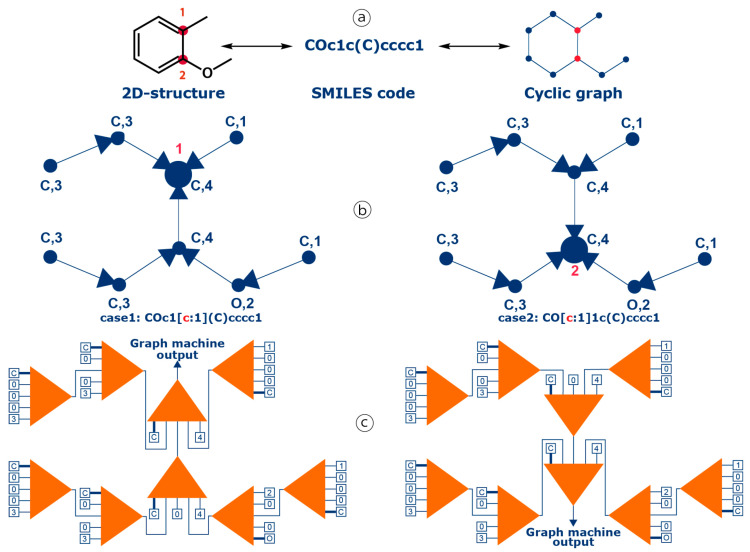
Graph-machine construction process for two carbons of 2-methoxytoluene: (ⓐ) conversion of the 2D structure of 2-methoxytoluene into a cyclic graph, (ⓑ) construction of the two directed acyclic graphs for the carbons marked in red, and (ⓒ) generation of the corresponding graph machines.

In graph-machine-based models, molecules are described as graphs derived from their 2D structure, and the parameterized functions (called graph machines) that compute the estimation of the property or activity of interest reflect the compound molecular structures. The procedure for graph-machine construction has been described in detail elsewhere [63,65,66]. In the present case, the main difference with previous descriptions of graph-machine design is that the property is computed for each benzenic carbon atom of all molecular structures, i.e., a graph machine is built for each carbon atom of interest. To this end, the SMILES code previously used to encode a molecular structure has been extended to point out to a specific atom of the molecule, so that the property estimate is computed for that atom. The algorithm has then been modified to perform this task routinely in the prediction step. On the contrary when building a training/validation dataset, an NMR expert’s help might be needed to attribute the chemical-shift values to the proper carbon atoms, and consequently to the proper SMILES codes. This construction process is illustrated in Figure 5 for two carbon atoms of 2-methoxytoluene (numbered 1 and 2) for which the ^13^C chemical shifts are estimated. The 2D molecular structure of 2-methoxytoluene, equivalent to the displayed SMILES code [67], is first converted into a cyclic graph (step ⓐ). In the next step, i.e., the transformation of the graph into a directed acyclic graph, the root node is assigned to one of the numbered atoms. This is performed by using SMILES codes containing special tags for the atoms of interest, COc1[c:1](C)cccc1 for atom #1 and CO[c:1]1c(C])cccc1 for atom #2. Two labeled-oriented acyclic graphs are consequently constructed, for which the designed output nodes map the carbons of interest, either #1 or #2 (large blue dots, step ⓑ). Finally, for these two directed acyclic graphs, a parameterized nonlinear function, called a *node function*, which is typically a multi-layer perceptron (MLP), with tanh activation functions for the hidden neurons and a linear output neuron, is implemented at each node of the graphs. The output computed by each node function (the orange triangles in Figure 5) is passed to the next node function, respecting the sequence of atoms in the initial molecule, and the graph orientation defined in step ⓑ. At the end, the function implemented on the node where all previous outputs converge, which is called the root node and corresponds to the carbon on interest, computes the output of the graph machine. The inputs of a node function are the node atom type, i.e., carbon or oxygen for 2-methoxytoluene, the degree of the atom, e.g., degree 4 for the two example atoms, and the outputs of the node function leaf nodes of the previous level. Since the two graphs have different output nodes, the two graph machines are also different (step ⓒ).

As a result, for a given benzenic carbon, the output of each graph machine depends solely on the structure of the molecule, the position of the carbon in the ring, and the parameters of the node function. In other words, it does not depend on any descriptor, with the carbon SMILES codes being the only required information. More details on graph-machine construction are provided in earlier papers [65,66].

### 3.2. ^13^C NMR Measurements for the Molecules of the Test Set

To assess the accuracy of the estimations with the selected graph-machine-based model described above, the ^13^C-NMR decoupled spectra of the 114 test molecules were recorded with a Bruker AC-300 advance at 100 MHz in deuterochloroform at 23 °C. Assignments of the chemical shifts to the proper carbon atoms were conducted, and in case of uncertainty between two carbon atoms for this attribution, other NMR techniques were used. As 92 molecules of the test set are also present in the SDBS ^13^C database, their measured ^13^C chemical shifts could be compared to the values retrieved from SDBS. While we did not notice any discrepancies in the shift assignments for the 472 benzenic carbon atoms present in these 92 molecules, some differences, mostly small, were observed for some shift values. The maximum difference is equal to 0.76 ppm in the case of the c-NO_2_ carbon of *o*-nitroanisole. The standard deviation of the 472 shift differences was then computed to get an idea of the expected accuracy of the estimations of the graph-machine-based model. This deviation, equal to 0.14 ppm, means that the results for the shift values thereinafter can be reported with one decimal digit. 

### 3.3. Model Selection

**Figure 6:**
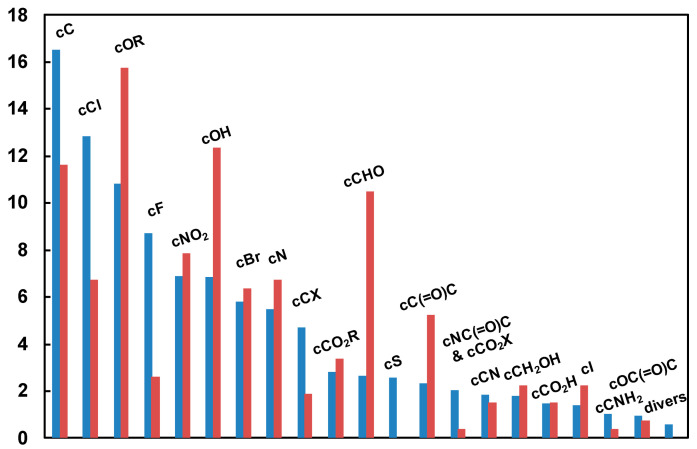
Distribution of functional carbons (as percentages) for the molecules of the training (blue bars) and test (bistre-colored bars) sets.

This step is especially important when designing machine-learning-based models. Its purpose is to find, given the data available for designing the model, the model complexity that will result in the best generalization: a model that is not complex enough is unable to fit the data, hence to generalize, while a model that is too complex (overparameterized) overfits the data and generalizes poorly. Basically, for graph machines, the number of adjustable parameters depends on the number of neurons present in the hidden layer of the multilayer perceptron (MLP) that has been used to design them. Therefore, the purpose of model selection is to find the complexity (number of hidden neurons) that results in the smallest estimation of the generalization error, given the data that are available for designing the model. To perform this task, the two previously defined sets of 8431 and 584 examples were, respectively, used as a training/validation set (called the training set for simplicity) for designing and selecting the model and as a test set for providing the final estimation of the generalization error of the selected model. The molecules of the test set were chosen so that (i) the distributions of molecules among the chemical families considered were similar in both sets for at least the most common functional groups, and (ii) the distribution of the ^13^C chemical-shift values was as uniform as possible on the range of measured values but depends also on the availability of the chemicals in the laboratory. The distributions of the functional carbon atoms for the two sets are shown in Figure 6. The distributions of the tertiary carbon atoms (CH) are not integrated in this figure for clarity, since they represent, respectively, 53% and 55% of the total number of carbon atoms in the two datasets.

As usual, for machine-learning-based models, the validity of the model is restricted to items that can be considered as realizations of random variables drawn from the same probability distribution as the examples of the training set. In practice, it means that reliable estimations can be expected for molecules that contain the same atoms as the molecules of the training set, and whose molecular structures are not too different from those present in the training set.

The first step is then to build the graph machines from the training-set examples and to train them. In the following, the set of graph machines that are constructed from the training examples will be termed the “graph machine-based model”. Given a training set of *N_T_* elements, the parameters (also termed weights) for these models are estimated by minimizing the sum of squared errors of the cost function *J*(***θ***) using the weight-sharing method between all nodes of all graph machines (Equation (2)):

(2) $$J(\theta )=\sum_{i=1}^{N_{T}}{\left(\delta_{exp.}^{i}-g^{i}(\theta )\right)}^{2}\;,$$

where $\delta_{exp.}^{i}$ is the measured value of the ^13^C chemical shift for the *i*-th element of the training set, ***θ*** is the vector of parameters, $g^{i}(\theta )$ is the value of the chemical shift estimated by the graph machine for that element, and *N_T_* = 8431, as detailed above. In this work, $g^{i}(\theta )$ is constructed as a combination of MLPs with a single hidden layer that reflects the graph structure of the *i*-th element. This MLP is a linear combination of nonlinear functions called hidden neurons, which are the hyperbolic tangent functions of a linear combination of the variables. All minimizations of the cost function are performed by the Levenberg–Marquardt algorithm, which is well suited to optimization problems with a moderate number of variables [68].

Once training is complete, the next step is to select the most appropriate model for predicting the chemical shifts of the test-set elements, i.e., to determine the complexity for which the generalization error is lowest. In the present study, the estimation of the generalization error for model selection is performed by the computation of the virtual leave-one-out (VLOO) score, which provides an unbiased estimation of the generalization ability of the model [69]. This strategy, to find the appropriate complexity of the graph-machine-based models, is chosen because the computation of the *VLOO score* is much faster than that of the LOO score while giving equivalent results [62]. The *VLOO score* is based on a first-order approximation of the estimation error that would have been observed on each example of the training set if that example had been withdrawn from that set before training. Thus, denoting by ***θ****_m_* the parameter vector after completion of training, the *VLOO score* is defined as the root mean square of the predicted errors (Equation (3)):

(3) $$VLOO\;score=\sqrt{\frac{1}{N_{T}}\sum_{i=1}^{N_{T}}{\left(\delta_{exp.}^{i}-{g^{i}(\theta }_{m}^{–i})\right)}^{2}}\;\;,$$

where ${g^{i}(\theta }_{m}^{–i})$ is a first-order approximation of the predicted chemical shift of carbon *i* provided by the *i*-th graph machine when the latter is not present in the training set (i.e., if the model had been trained on all training set carbons except carbon *i*), and $\delta_{exp.}^{i}$ is the measured value of the chemical shift for the *i*-th carbon of the training set. In the present case, the VLOO score is computed for the 8431 carbons of the dataset. For each complexity, 100 trainings are performed with different initial parameter values, and the mean and standard deviation of the 25 smallest VLOO scores are computed for the selection of the most appropriate complexity. In general, as complexity increases, the VLOO score reaches a floor value, unless overfitting occurs, in which case the VLOO score increases after this value. The optimal complexity is that for which this minimum score is reached.

After selecting the appropriate complexity of the graph-machine-based model, the parameter vectors after training ***θ***_m_ for the 25 models that have the smallest VLOO score values are stored. These selected models are then used to predict the carbon chemical shifts of the 122-molecule test set. For all 584 carbons, graph machines are constructed as explained above (e.g., six graph machines for the six benzenic carbons of 2-methoxytoluene shown in Figure 5), and the ***θ***_m_ parameters of the 25 kept models are successively assigned to their node functions. Finally, the average of the resulting 25 graph-machine outputs provides an estimate of the chemical shift for each of the 584 carbons. The true benefit of this approach is the absence of descriptors; the SMILES codes are the only required information. Moreover, the same set of graph machines can be reused for estimating the same property in different conditions, e.g., if compounds are dissolved in another solvent. All it takes is to learn the new chemical-shift values.

## 4. Conclusions

The estimation of the ^13^C chemical shifts of organic compounds still attracts much attention due to the importance of that property in the spectroscopic area. The present article reports four main innovations: (i) the estimation of the ^13^C chemical shifts of benzenic carbons by graph machines, a machine-learning method that allows the estimation of properties or activities of molecules directly from their structure described by their SMILES codes, without requiring any other descriptors; (ii) the graph-machine method, applied to a set of 10577 carbons, estimates their chemical shift with a root-mean-square error of 0.6 ppm; (iii) the comparison of the accuracy of shift predictions obtained by several methods (ChemDraw, MestReNova, ACD, NMRshiftDB, and graph machines); and (iv) a software (v. 1.0) that is available for download to predict the ^13^C chemical shifts of a benzenic molecule from its SMILES code.

A database of 8431 benzenic ^13^C chemical shifts is used for training and model selection, and a database of 584 benzenic ^13^C chemical shifts is used for testing. Graph machines, which perform regression from the graphs derived from the SMILES codes, are first constructed for each carbon. The graph-machine-based models are trained, and a model selection is performed by virtual leave one out (VLOO) to select a node function complexity of 26 neurons. The resulting root-mean-square error on the test set using this complexity is then equal to 0.7 ppm.

After analysis of the estimation and prediction results, a final graph-machine-based model, with the same complexity as the previous model, is built and trained on a large set of 10557 ^13^C experimental shifts compiled and checked carefully. Successfully tested on a set of 156 carbons of 28 molecules gathered from freshly published data, this model is applied to the ^13^C shift prediction of a larger set of 1011 carbons from 171 benzenic molecules, which contain up to 10 different elements other than carbon. Its performance is then compared with that of several commercial software packages. While a root-mean-square error of 0.9 ppm is obtained with graph machines for the prediction of the shift of these 1011 carbons, Chemdraw, MestReNova, and ACD lead to values of 3.4, 1.9, and 1.8 ppm, respectively.

The main limitations of the graph-machine approach are not very different from those of conventional neural networks. Molecules derived from benzene for which the chemical shift of the ring carbons is to be estimated must contain functionalities that have been encountered in the training set. If new atoms or conformational effects are present in its structure, the predicted shifts will not be reliable and will have to be handled with care.

In any case, the current results demonstrate for the first time the ability of graph machines to accurately estimate an atomic property, such as the chemical shift of a carbon atom, from the 2D structure of the molecule. This is consistent with the fact that this property strongly depends on the neighborhood of the atom under consideration and shows that the information contained in the molecule’s SMILES code is then sufficiently relevant. In the same way, as for the carbon atom, graph machines are effective for predicting the chemical shift of the proton, as well as that of other NMR-active nuclei. In a future article, we will show that the use of graph machines is also relevant for predicting another atomic property, namely the pKa of an acid group in a molecule. To model this property, however, we need to take into account the hydrogen atoms responsible for the acidity of the molecules whose pKa(s) we are studying, which graph machines can perform without difficulty.

For easy duplication of the presented results and testing of the method on other carbons belonging to similar molecules, as those present in our database, demonstration software (v. 1.0) is made available in the Supporting Information, Sections S3 and S4.

## Figures and Tables

**Table 1:** RMSE (in ppm) computed with 6 models for 22 benzenic structures.

Model Algorithm	Nmrdb HOSE	NMRshiftDB HOSE [NN] ^2^	Nmr Predict HOSE + NN	ChemDraw Increments	MestReNova Ensemble	ACD HOSE + NN
RMSE (ppm) ^1^	4.7	6.6 [1.1]	3.8	3.3	2.2	1.9

^1^ The root-mean-square error is computed for the 128 benzenic carbons in the set of 22 molecules. ^2^ Only 78 chemical shifts are computed with the NN algorithm, as Br and I atoms are not allowed.

**Table 2:** Estimation of the chemical shifts from SMILES of the 8431 carbons of the training set by graph-machine-based models of increasing complexity.

Number of Hidden Neurons ^1^	14	16	18	20	22	24	26	28	30
RMSTE ^2^	1.08	0.97	0.86	0.79	0.73	0.67	0.63	0.58	0.55
VLOO score ^3^ (ppm)	1.20 (0.003)	1,08 (0.008)	0.99 (0.002)	0.92 (0.001)	0.87 (0.002)	0.82 (0.003)	0.78 (0.004)	0.75 (0.005)	0.72 (0.001)
Computation time ^4^	0.9	1.1	1.3	1.7	2.1	2.6	3.2	4.1	5.0

^1^ Results for a number of hidden neurons equal to 4–12 are given in Table S2 of the SI. ^2^ RMSTE value in ppm of the trained model (out of 100) having the smallest RMSTE for the 8431 carbon chemical shifts of the training set. ^3^ Mean and standard deviation (in parenthesis) of the VLOO scores (defined in Section 3.3) averaged over the 25 trained models (out of 100) having the smallest VLOO scores computed for three different parameter initializations for the 8431 carbons of the training set. ^4^ Average time (in seconds on an iMacPro) to compute one carbon chemical shift of the test set using the 25 trained models (out of 100) having the smallest VLOO scores.

**Table 3:** Performance of the GM26 model for the training and test sets.

Dataset	$N_{T}$ ^1^	RMSE ^2^	MAE ^2^	R ^2,3^	MIN ^4^	MAX ^5^
Training	8431	0.5	0.4	0.998	−3.3	3.7
Test	584	0.7	0.5	0.997	−3.8	4.1

^1^ Number of carbons in datasets, ^2^ RMSE, and MAE (mean absolute error) in ppm, are averaged over the 25 trained models (out of 100) having the smallest VLOO scores for the *N_T_* carbons of the training set, ^3^ determination coefficient of the scatter plot of estimated versus measured shifts for the carbons of the dataset, and ^4,5^ minimum and maximum deviations from experiment, in ppm.

**Table 4:** Cases assigned to benzenic carbons explaining the large deviations observed for their estimated chemical shifts, and recommendations for improvement.

Benzenic Carbon ^1^	*δexp.* ^2^	*δest.* ^2^	Δ*δ* ^3^	Cases	Recommendations
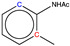	131.2 124.7	128 121.4	+3.1 +3.3	i i	take *δ_exp_*_._ = 129.4 take *δ_exp_*_._ = 123.6
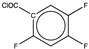	113.9	117.2	−3.3	i	take *δ_exp_*_._ = 118.3
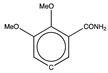	124.3	121.3	+3	ii or iv	keep value
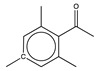	138.2	141.5	−3.3	iv	add 2,6-dimethyl- acetophenones
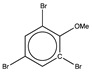	117.5	114.3	+3.2	iv	add 2,6-dibromoanisoles
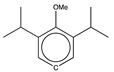	124.6	121.5	+3.1	iv	add 2,6-di-tert- butylanisoles
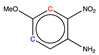	106.3 126.7	109.4 123	−3.1 +3.7	iv	remove 2-nitro-*p*-anisidine
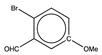	155.6	159.4	−3.8	i	take δ_exp._ = 159.3
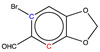	113.2 121.6	109.1 118.4	+4.1 +3.2	i iv	take δ_exp._ = 108.1 add 2-bromo- benzaldehydes
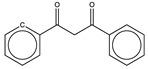	128.7	131.7	−3	iii	add 1,3-diketones
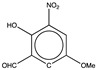	122.9	119.5	+3.4	i	add 3-methoxy- 5-nitrobenzaldehyde

^1^ Benzenic carbons in the structures shown are indicated by a C, marked in blue and red if 2 carbons are present in the same molecule. The first block corresponds to training set molecules, and the second block to test set molecules. ^2^ The red and blue colors correspond to the shift for red and blue carbons respectively. ^3^ Differences in ppm between measured shift $\delta_{exp.}$ and predicted shift $\delta_{est.}$.

**Table 5:** Examples of molecules with new atoms or functionalities used for training.

Functionality or Atom in the Substituent	Number of Molecules	Example of Benzene Substituent
Phenoxy	30	*p*-CH(=O)C_6_H_4_O
1,3-diketone	14	C_6_H_5_C(=O)CH_2_C(=O)
Sulfoxide	17	H_3_CS(=O)
Acetic acid	4	HO_2_CCH_2_
Acetonitrile	9	NCCH_2_
Benzoyl	7	C_6_H_5_C(=O)
Azide	28	N_3_
Crowded carbon	35	*t*-Bu in position 2,4,6
P	34	C_6_H_5_OPH(=O)O
Si	21	Me_2_SiH

**Table 6:** Performance of the new GM26 model for the training and test sets.

Dataset	$N_{T}$ ^1^	RMSE ^2^	MAE ^2^	R^2^	MIN ^3^	MAX ^4^
Training	10,577	0.6	0.4	0.997	−3.5	3.6
Outliers	12 ^5^	2.0 (3.3) ^6^	1.6	0.986	−2.5	2.6
Test	156	1.0	0.7	0.995	−3.4	5.0

^1^ Number of carbons in datasets, ^2^ RMSE and MAE, in ppm, averaged over the 10 trained models (out of 100) having the smallest VLOO scores for the N*_T_* carbons of the training set, ^3,4^ minimum and maximum deviations from the experiment in ppm, ^5^ for the 10 outliers, which are all members of the present training set, only the 12 ‘faulty’ shifts are considered, and ^6^ RMSE in parenthesis corresponds to the results given in Section 2.4.

**Table 7:** Comparison of the performance of the new GM26 model and four other models on a test set of 1011 ^13^C chemical shifts.

Model	RMSE	MAE	R^2^	MIN ^2^	MAX ^3^	No C ^4^
GM26	0.9 ^1^	0.7 ^1^	0.997	−3.6	3.6	9
ChemDraw	3.4	2.2	0.956	−17.2	27.6	256
MestReNova	1.9	1.4	0.986	−10.1	9.5	103
ACD	1.8 ^5^	1.2	0.988	−8.4	8.5	95
NMRshiftDB (NN)	1.1 ^5^	0.8	0.995	−4.5	4.3	15

^1^ RMSE and MAE, in ppm, averaged over the 10 trained models (out of 100) having the smallest VLOO scores for the 10577 carbons of the training set, ^2,3^ minimum and maximum deviations from experiment in ppm, ^4^ number of carbons with a predicted shift deviation greater than 3 ppm in absolute value, and ^5^ for ACD and NMRshiftDB, the RMSEs are computed with 996 and 596 shift values respectively.

## Data Availability

The data presented in this study are available in article and Supplementary Materials.

## Supplementary Materials

The following supporting information can be downloaded at https://www.mdpi.com/article/10.3390/molecules29133137/s1: The list of compounds used in the present work for training and testing of the graph-machine models, and the list of compounds of the datasets used for comparison are available online as excel files, under the name of CS128-SI.xlsx for Table S1: “Carbon names, carbon SMILES, experimental, Nmrdb-estimated, NMRshiftDB-estimated, NMRshiftDB_NN-estimated, NmrPredict-estimated, Chemdraw-estimated, MestReNova-estimated and ACD-estimated ^13^C chemical shift values in ppm for the set of 22 molecules”, ComplexitySelection-SI.xlsx for Table S2: “Estimation of the chemical shifts from SMILES by graph machine based models of increasing complexity”, CS8431A-584T-SI.xlsx for Table S3: “Carbon names, carbon SMILES, experimental and GM26-estimated ^13^C chemical shift values in ppm for the training set of 1637 molecules”; Table S4: “Carbon names, carbon SMILES, experimental and GM26-estimated ^13^C chemical shift values in ppm for the test set of 114 molecules”, Anisidine-SI.xlsx for Table S5: “Carbon names, carbon SMILES, experimental, graph machine-estimated, Gaussian-estimated, ACD-estimated, MestReNova-estimated, Chemdraw-estimated, NMRshiftDB-estimated and NmrPredict-estimated ^13^C chemical shift values in ppm for 2-nitro-*p*-anisidine”, CS10577AConstruction-SI.xlsx for Table S6: “Names and references of the 281 added molecules to form the final 10577-carbon training set”; Table S7: “Names, SMILES, and explanation for deletion of six molecules in the 1637-molecule training set”, CS10577A-156T-1011T-SI.xlsx for Table S8: “Carbon names, carbon SMILES, experimental and GM26-estimated ^13^C chemical shift values in ppm for the training set of 2026 molecules”; Table S9: “Carbon names, carbon SMILES, experimental and GM26-estimated ^13^C chemical shift values in ppm for the test set of 28 molecules”; Table S10: “Carbon names, carbon SMILES, experimental, GM26-predicted, MestReNova-estimated, ACD-estimated, Chemdraw-estimated and NMRshiftDB-NN-estimated ^13^C chemical shift values in ppm for the test set of 171 molecules”, MoleculeLists-SI.xlsx for Table S11: “Names and references of the 22 molecules for the 128-carbon set”; Table S12: “Names of the 1637 molecules of the 8431-carbon training set”; Table S13: “Names of the 114 molecules of the 584-carbon test set”; Table S14: “Names of the 2026 molecules of the 10577-carbon training set”; Table S15: “Names and references other than SDBS for the 28 molecules of the 156-carbon test set”; Table S16: “Names and references of the 171 molecules of the 1011-carbon test set” A few reminders about carbon-13 nuclear magnetic resonance, analysis of GM26 chemical-shift estimates on 8431-training and 584-test sets, GM demonstration with docker containers, and GM results with Docker are available as a pdf file under the name CSdemo-SI. The test data used in the demo can be found in an Excel file named Test156-SI. Figure S1. ^13^C spectrum of 2-methoxytoluene recorded at 101 MHz in a CDCl_3_ solution; carbon chemical-shift values are in ppm. Figure S2. Structure of training-set molecules with at least one carbon whose shift estimation with the GM26 model shows an absolute deviation greater than 3 ppm. Experimental and estimated chemical-shift values ($\delta_{exp.}$ and $\delta_{est.}$ in ppm) are for carbon atoms shown in red and blue. Figure S3. Comparison of extrapolated chemical-shift values for carbons 5 and 6 of 2,3-dimethoxybenzamide, computed from experimental values for relevant shifts of 2-methoxybenzamide, with their measured values. Experimental, estimated, and extrapolated (in red) chemical-shift values ($\delta_{exp.}$, $\delta_{est.}$, and $\delta_{extr.}\;$ in ppm) refer to numbered carbon atoms. Figure S4. Comparison of extrapolated chemical-shift values for carbons 3 and 5 of 2-nitro-p-anisidine, calculated from experimental values for relevant carbon shifts of 2-nitroaniline (①), p-anisidine (②), and 3-nitroanisole (③) with their measured values. Experimental, estimated, and extrapolated chemical-shift values ($\delta_{exp.}$, $\delta_{est.}$, and $\delta_{extr.}\;$ in ppm) refer to numbered carbon atoms. Figure S5. Structure of the test-set molecules possessing at least one carbon whose shift prediction with the GM26 model has an absolute deviation greater than 3 ppm. Experimental and predicted chemical shifts ($\delta_{exp.}$ and $\delta_{pred.}$ in ppm) are for carbon atoms shown in color. Figure S6. Structures of major enol form of 1,3-diphenyl-1,3-propanedione with a stabilizing H-bond, 5-methoxy-3-nitrosalicylaldehyde with an intramolecular H-bond and 3-methoxy-5-nitrobenzaldehyde; the carbon atoms mapped for the enol structure, e.g., 1 and 1′, are those that are equivalent in the NMR spectrum. The carbons shown in red are those whose chemical shift is difficult to predict. References [70,71,72,73,74,75,76,77,78,79,80,81,82,83,84,85,86,87,88,89,90] are in Supplementary Materials.
