# Supplementary material for: Can Graph Machines Accurately Estimate 13C NMR Chemical Shifts of Benzenic Compounds?
Source: Molecules. 2024 Jul 1;29(13):3137. doi: 10.3390/molecules29133137 (PMC11243075; doi:10.3390/molecules29133137)
Supplement: Supplementary file 1 [file molecules-29-03137-s001.zip › CSdemo-SI.pdf]

Supporting Information

Can Graph Machines accurately estimate ^{13}C NMR chemical shifts of benzenic compounds?

François Duprat*, Jean-Luc Ploix, Gérard Dreyfus

Chimie Moléculaire, Macromoléculaire, Matériaux, ESPCI Paris, PSL University, 10 rue Vauquelin, 75005 Paris, France; jean-luc.ploix@espci.psl.eu, gerard.dreyfus@espci.psl.eu

* Correspondence: arthur.duprat@espci.psl.eu

S1. A FEW REMINDERS ABOUT CARBON-13 NUCLEAR MAGNETIC RESONANCE

Carbon-13 NMR basics

NMR is a physical phenomenon based on the quantum-mechanical magnetic properties of an atom's nucleus. NMR-active nuclei (with non-zero magnetic moment), such as ^{1}H and ^{13}C, are capable of absorbing radio frequencies when placed in a strong magnetic field. When this absorption occurs, the nucleus is said to be in resonance. Since different atoms (even if of the same kind, e.g. ^{13}C) within a molecule resonate at different frequencies in a magnetic field of given strength, the observation of the resonance frequencies provides information about its structure. The quantity used to quantify this phenomenon is called chemical shift (δ). It is expressed in parts per million (ppm) by frequency, and is defined by Equation (S1), where the numerator is expressed in Hz and the denominator in MHz:

$$\delta = \frac{\nu_{mol} - \nu_{ref}}{\nu_0} \quad \text{(S1)}$$

ν_{mol} is the resonance frequency of an atom in the molecule under study, ν_{ref} is the resonance frequency of a reference compound and ν_0 is the frequency of the spectrometer. The detected frequencies for ^{1}H and ^{13}C nuclei in a given solvent are usually referenced against tetramethylsilane (TMS), for which δ_{TMS} is zero.

The chemical shift is one of the most important characteristics of a nucleus in terms of NMR. The advantage of using this atomic property lies in its independence from the frequency of the spectrometer used. For example, the ^{13}C spectrum of 2-methoxytoluene, either recorded at frequencies of 25.2 [1] or 101 MHz [2] in deuteriochloroform ($CDCl_3$) with the same reference (TMS), leads to the chemical shifts δ_{exp} (in ppm) assigned to the carbons of the structure shown in Figure S1 [2].

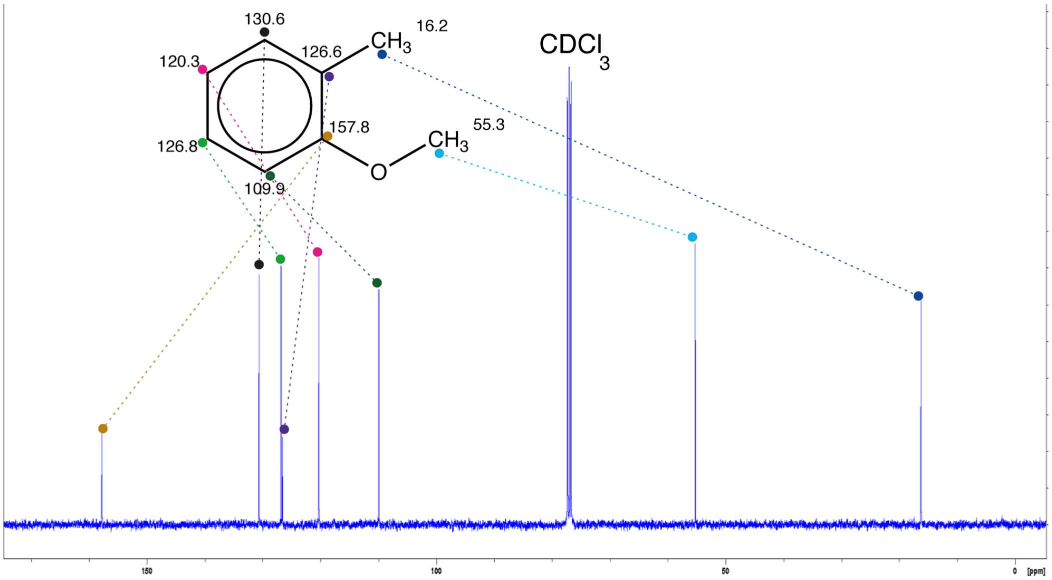

Figure S1. ^{13}C spectrum of 2-methoxytoluene recorded at 101 MHz in a $CDCl_3$ solution; carbon chemical shift values are in ppm.

As the chemical shift of an individual carbon atom depends on its hybridization state and the overall electronic environment surrounding the nucleus (its atomic properties), its value might differ substantially for carbons in the same molecule. Indeed, some carbon chemical shifts are sensitive to the effects of substituents, sometimes up to several bonds away from the resonating carbon, particularly in conjugated or aromatic systems. Thus, the carbon of the methoxy group with the light blue dot in Figure S1 has a chemical shift equal to 53.3 ppm, very different from that of the benzylic methyl (16.2 ppm, dark blue dot) or the quaternary carbon attached to the benzylic methyl (126.6 ppm, dark purple dot). The resulting high deshielding (displacement to high frequency) of the latter atom is due to the fact that it belongs to a benzene ring. This phenomenon is observed for all ring carbon atoms, which resonate above 100 ppm. Interpreting such a spectrum, starting with the attribution of all the chemical shifts observed, is part of the aforementioned elucidation work. And this can be extremely difficult in the case of complex molecules or if the structure of the molecule under study is not known accurately. It is then essential to use algorithms that are capable of providing chemical shift predictions and also very useful for a whole range of applications, such as structure validation [3], automatic assignment [4] and compound elucidation [5].

Estimation of ^{13}C chemical shifts using increments

Consequently, spectroscopists sought to establish in the early sixties empirical relationships between molecular structures and chemical shifts, in order to estimate unknown chemical shift values accurately [6,7]. The first models that were developed for the estimation of ^{13}C NMR chemical shifts were based on a general additive scheme using linear regression [8]. In such methods, the basic idea is to introduce, for a given carbon of a molecule in which a hydrogen —not necessarily on that carbon— has been replaced by a substituent *i*, an additional term δ_i (the so-called increment) corresponding to the presence of that substituent. δ_i values are determined by recording the chemical shift differences between the unsubstituted (δ_0) and singly substituted (δ_{0i}) molecule according to Equation (S2):

$$\delta_i = \delta_{0i} - \delta_0 \qquad \text{(S2)}$$

In the presence of multiple substituents *i* the chemical shift of the carbon under consideration, δ_C, would be calculated by summing the values of all δ_i increments, applying the following formula, in the case of an *i*-substituted benzenic compound (Equation (S3)):

$$\delta_C = \delta_{benzene} + \sum_i \delta_{0i} + \mathrm{K} \quad \text{(S3)}$$

where K is a constant representing a correction for mutual steric and/or electronic interactions of nearby substituents [9,10]. For 2-methoxytoluene (Figure S1), the chemical shift of the ring carbon bearing the methoxy group (157.8 ppm, brown dot) is calculated, in deuterochloroform, as follows:

$\delta_{COMe} = \delta_{Bz} + \delta_{ipso(OMe)} + \delta_{ortho(CH_3)} + \mathrm{K} = 128.5 + 33.5 + 0.7 - 4.1 =$ 158.6 ppm, in which δ_{Bz} is the ^{13}C chemical shift of a carbon in benzene, $\delta_{ipso(OMe)}$ represents the increment due to the *ipso* effect of the methoxy group (OMe) on the benzenic carbon cOMe, and $\delta_{ortho(CH_3)}$ that of the *ortho* effect of the methyl group. The K correction is not negligible in this equation, as the two substituents are ring neighbors. In this example and for this carbon, the estimated value is close to the experimental value (deviation 0.8 ppm).

S2. ANALYSIS OF GM26 CHEMICAL SHIFT ESTIMATES ON 8431-TRAINING AND 584-TEST SETS

Analysis of Training Set Estimations with the largest errors

For the training set, seven molecules (structures shown in Figure S2) have an estimated shift value, for at least one of their carbons, which differ from the experimental value by more than 3 ppm (0.4 %). The possible reasons for this discrepancy are analyzed and linked to cases i–iv: (i) the measured chemical shift value is incorrect for various reasons, e.g. the sample solvent is dimethylsulfoxide instead of $CDCl_3$, (ii) the experimental shift has been wrongly attributed to a given carbon, which most often corresponds to an inversion in the assignment of shifts between two carbon atoms, (iii) the structure used to generate the SMILES does not match the sample form present in solution, and (iv) the model cannot learn a specific structural feature of the molecule, which is often the case if this feature is poorly represented in the training set. For 2'-methylacetanilide and 2,4,5-trifluorobenzoyl chloride, different values are published in the literature for the carbon chemical shifts marked in red and blue, so this is case i. The carbons of 2'-methylacetanilide, whose shifts are announced at 124.7 and 131.2 ppm in SDBS [1], were thus measured at 123.6 and 129.4 ppm respectively in four publications [11–14]. Likewise, the shift of the carbon bearing the main function of 2,4,5-trifluorobenzoyl chloride reported at 113.9 ppm in SDBS [1], is published at 118.3 ppm by Sigma-Aldrich [15], and also predicted at 118.5 ppm by ACD software [15]. It would therefore be advisable for these two molecules to correct the experimental shift values for future use.

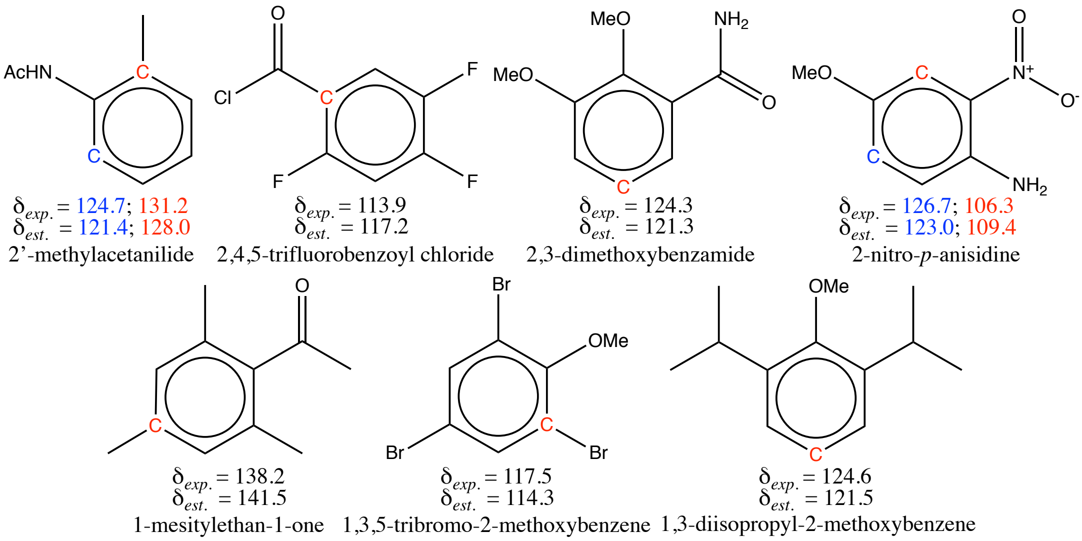

Figure S2. Structure of training set molecules with at least one carbon whose shift estimation with the GM26 model shows an absolute deviation greater than 3 ppm. Experimental and estimated chemical shifts values ($\delta_{exp.}$ and $\delta_{est.}$ in ppm) are for carbon atoms shown in red and blue.

2,3-dimethoxybenzamide (third structure in the first row of Figure S2) could correspond to case iv, since intramolecular hydrogen bonding, not encoded in its SMILES, may exist in solution. Indeed, its proton spectrum contains two peaks attributed to the amido group protons [16], one of them being deshielded due to the establishment of a hydrogen bond. Such a bond is visualized as dotted lines in the structure of 2,3-dimethoxybenzamide shown in the Figure S3. Moreover, other 2,3-dimethoxybenzamide derivatives exhibit hydrogen bonding in the solid state or in solution [17,18]. As a result, the presence of this electrostatic bond can modify the transmission of electronic effects from substituents in the benzene ring, which might not be captured properly by the GM model. But the discrepancy in shift (3.1 ppm) may also result from a misassignment of peaks for carbons numbered 5 and 6 in the 2,3-dimethoxybenzamide structure shown in Figure S3 (case ii). Consider the 2-methoxybenzamide molecule (first structure of Figure S3), whose proton spectrum shows the same phenomenon of non-equivalence between the amide group protons due to the presence of a similar hydrogen bond. Assuming that a methoxy group is added in position 3 of this structure (central structure in Figure S3) to obtain the 2,3-dimethoxybenzamide structure, and knowing that a methoxy group induces in the *meta* and *para* positions of a benzene ring, a deshielding of 1.0 ppm and a shielding of 7.8 ppm respectively [19], the extrapolated shifts calculated from the experimental shift values of 2-methoxybenzamide [20] would be equal to 122.2 ppm for carbon no. 5 and 124.8 ppm for carbon no. 6. It should be noted that this approximate computation does not include any corrective terms, as shown by equation S3. This result is the opposite of $\delta_{exp.}$ equal to 124.3 and 123 ppm for the shifts of carbon numbered 5 and 6 found in SDBS [1], but the extrapolated result for carbon no. 5 ($\delta_{extr.}$ = 122.2 ppm) would be more in line with the value estimated by the model ($\delta_{est.}$ = 121.2 ppm), with an expected deviation of around 2 ppm instead of 3.1 ppm, in the event of re-training. As a result, the shift assignments of carbon numbered 5 and 6 in 2,3-dimethoxybenzamide could be exchanged. A DFT calculation could remove the ambiguity concerning the assignment of shifts for these two carbons.

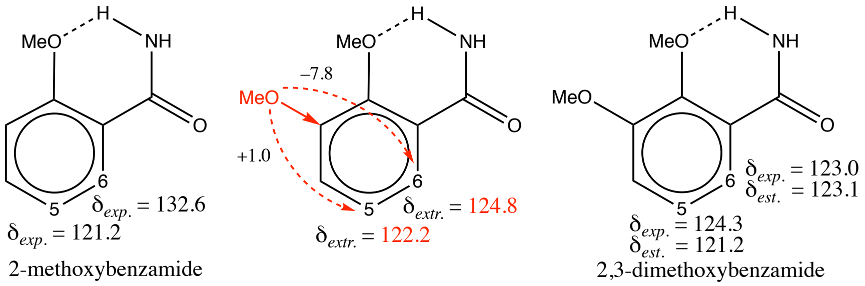

Figure S3. Comparison of extrapolated chemical shift values for carbons 5 and 6 of 2,3-dimethoxybenzamide, computed from experimental values for relevant shifts of 2-methoxybenzamide, with their measured values. Experimental, estimated and extrapolated (in red) chemical shifts values ($\delta_{exp.}$, $\delta_{est.}$ and $\delta_{extr.}$ in ppm) refer to numbered carbon atoms.

All molecules whose structures are shown in the second row of Figure S2, correspond to case iv. They all have a substituent on the ring, which is surrounded by bulky neighbors, preventing the regular transmission of electronic effects in the benzene system. For example, 1-mesitylethan-1-one (second row, first molecule in Figure 3) cannot accommodate the acetyl group ($COCH_3$) in the ring plane, due to the methyl group present on neighboring ring carbons. Consequently, the graph machine model estimates a higher chemical shift value than that observed for the carbon in position 4 (in red), because it takes into account the standard deshielding effect of the acetyl group, whereas it is significantly reduced. For all three molecules, few similar examples are present in the training set, preventing the model from efficiently learning their features. Therefore, as indicated above, the addition of similar molecules to a future training set is desirable. 2-nitro-*p*-anisidine, whose structure is the last in the first row of Figure S2, is a tough case, with two chemical shifts poorly estimated. These shifts are also predicted with a large discrepancy by all software programs we have used, except Gaussian 09 (see Table S5 in the SI file 'Anisidine-SI.xlsx' for more details). In an attempt to understand the underlying effects of substituents in 2-nitro-*p*-anisidine, the expected shift values for its carbons numbered 3 and 5 (central structure in Figure S4) are calculated,

starting from molecules that already have two substituents of 2-nitro-*p*-anisidine in the appropriate position. To this end, the experimental shifts of 2-nitroaniline (①), *p*-anisidine (②) and 3-nitroanisole (③) for carbons numbered 3 and 5 or 2 and 6 are incremented with the values corresponding to the added substituents, i.e. methoxy, nitro and amino respectively, as shown in Figure S4.

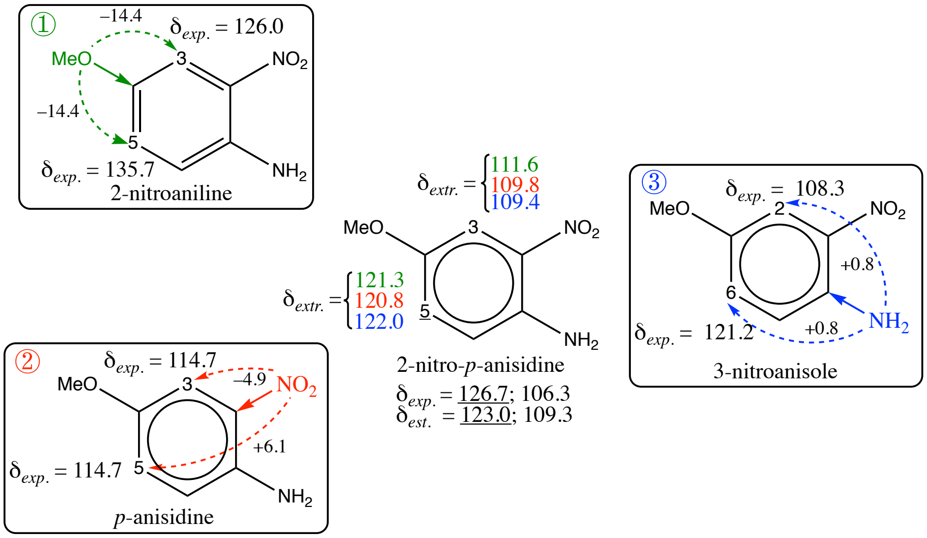

Figure S4. Comparison of extrapolated chemical shift values for carbons 3 and 5 of 2-nitro-*p*-anisidine, calculated from experimental values for relevant carbon shifts of 2-nitroaniline (①), *p*-anisidine (②) and 3-nitroanisole (③) with their measured values. Experimental, estimated and extrapolated chemical shifts values ($\delta_{exp.}$, $\delta_{est.}$ and $\delta_{extr.}$ in ppm) refer to numbered carbon atoms.

Interestingly, all carbon shifts for the framed molecules in Figure S4 are accurately estimated (③) or predicted (① and ②) by the GM model (standard deviation (SD) 0.3 ppm for 16 carbon shifts, maximum deviation 0.6 ppm). However, as soon as a third substituent is virtually added to each of them (methoxy, nitro and amino respectively) to have the 2-nitro-*p*-anisidine structure, the extrapolated shifts $\delta_{extr.}$ resulting from the addition of the appropriate increments to the measured shifts of carbons numbered 3 and 5 (① and ②) or 2 and 6 (③) are fairly consistent, but more than 3 ppm away from the experimental carbon shifts of 2-nitro-*p*-anisidine (109.5-111.5 instead of 106.3 and 120.9-122 instead of 126.7 in ppm). The extrapolated values closest to the measured shifts are those obtained in case ③, which is consistent with the fact that the added amino substituent in the meta position of the carbons concerned has a low increment value (+0.8). One hypothesis to explain the values observed for carbon 3 and 5 shifts in 2-nitro-*p*-anisidine, and compatible with the effects of substituents on both positions, would be to credit the nitro group in this particular case with greater shielding effect in the *ortho* position (−8.4 instead of −4.9) and a strong deshielding effect in the *para* position (+12 instead of +6.1). This anomaly could also be explained by the existence of an intramolecular hydrogen bond between the neighboring nitro and amino groups of 2-nitro-*p*-anisidine, but this is not confirmed in its proton NMR spectrum at the acquisition (room) temperature [21], moreover this H-bond would also be present for 2-nitroaniline (①), which is not the case for its proton spectrum either. Finally, it should be pointed out that the estimated shifts of the corresponding carbons for the 2-nitroaniline derivatives present in the training set, namely 4-fluoro-2-nitroaniline, 2-nitro-4-(trifluoromethyl)aniline and 2-nitro-*p*-toluidine, show a smaller deviation, below 1.5 ppm at most. As no convincing explanation could be provided for the discrepancies observed for 2-nitro-*p*-toluidine, for which the shifts measured in the laboratory have been validated in many references, it would be wise to remove it from the training set. Other 2-nitroaniline derivatives also possessing a substituent located *para* to the amino group, with the same electronic effects as those of the methoxy group, could also be added for further investigation.

Analysis of Test Set Predictions with absolute errors above 3 ppm

Four molecules in the test set have at least one carbon with an absolute chemical shift deviation greater than 3 ppm (3.5 %). Their structures are shown in Figure S5, with carbons that exhibit a high deviation in

their estimated shift drawn in color. Equivalent arguments involving the cases i–iv listed in the previous section can be drawn upon for all carbons in the test set whose shift prediction is in error. Thus, the significant prediction errors observed for two of the carbon atoms indicated in red in the structures in the first row of Figure S5 result from the use of erroneous experimental values (case i). The experimental shift of 2-bromo-5-methoxybenzaldehyde faulty carbon was recorded for our sample at 155.6 ppm, but several authors have published [22–24] its shift value equal to 159.3 ppm, which is more in line with the GM26 prediction. We have not been able to explain this difference in measured values for a single peak, but have sometimes observed the same phenomenon for other molecules for which several spectra recorded under the same conditions have been published. Likewise, a small peak with $\delta_{exp.}$= 113.2 ppm was mistakenly assigned to the red tertiary carbon of 6-bromopiperonal in Figure S5, whereas its true chemical shift value is 108.1 ppm. These values must be corrected for future use.

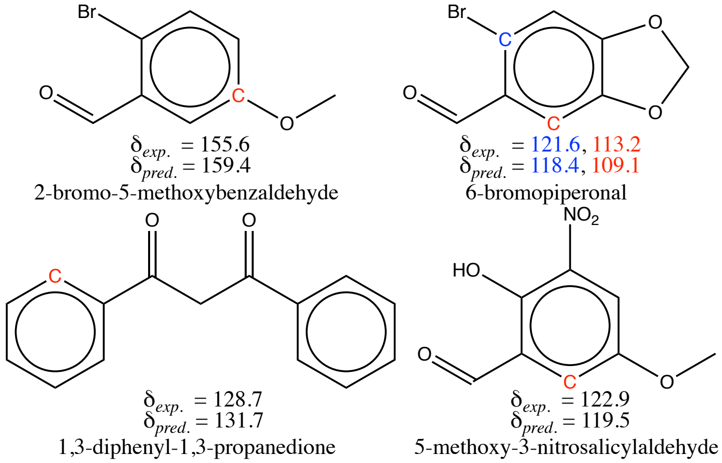

Figure S5. Structure of the test set molecules possessing at least one carbon whose shift prediction with the GM26 model has an absolute deviation greater than 3 ppm. Experimental and predicted chemical shifts ($\delta_{exp.}$ and $\delta_{pred.}$ in ppm) are for carbon atoms shown in color.

For molecules whose structures are shown in the second row of Figure S5, the large error could correspond to the third hypothesis (case iii). In fact, both molecules exhibit intramolecular hydrogen bonding in $CDCl_3$ solution, as confirmed by the observation of an unshielded line in their proton NMR spectrum at 17.0 and 10.9 ppm respectively [25,26]. The former value, well above 12 ppm, indicates a particularly strong hydrogen bond, which can be explained by the predominance, in solution, of the tautomeric enol form of 1,3-diphenyl-1,3-propanedione shown in Figure S6 (first structure) [25]. This predominance is confirmed by the presence in the same spectrum of a tiny peak at 4.65 ppm, a shift value expected for the methylene group protons of the dione form. The (*Z*)-3-hydroxy-1,3-diphenylprop-2-en-1-one structure should then be used as input for the GM construction instead of the dione's, and the estimated shifts for the four ring carbons (two are equivalent) should result from averaging the estimated values computed for the matching carbons of the two rings (carbons labelled in Figure S6). There is no point in carrying out such a calculation at present, as no benzene derivatives with enol-type substituents are present in the training set. For 5-methoxy-3-nitrosalicylaldehyde, whose structure is also shown in Figure S6, we cannot explain such a large chemical shift deviation (3.4 ppm) for the tertiary carbon adjacent to the CHO group, despite the presence of an H-bond as indicated. In fact, all 12 salicylaldehyde derivatives present in the training set have an H-bond, and all the chemical shifts of the *ortho*-aldehyde carbon are estimated by the GM model with a smaller deviation (maximum deviation 1.9 ppm, mean 1.0 ppm and SD 0.4 ppm). The same applies to the six salicylaldehyde derivatives of the test set, but with higher deviations (maximum deviation 1.9 ppm, mean 1.6 ppm, SD 0.3 ppm). It is also possible that, as with 2-bromo-5-methoxybenzaldehyde mentioned above, the shift value recorded for the indicated tertiary carbon of 5-methoxy-3-nitrosalicylaldehyde is incorrect, which cannot be verified since no reference is available in the literature. Meanwhile, chemical shift prediction with the GM26 model of the labeled carbon for 3-methoxy-5-nitrobenzaldehyde (Figure S6), not present in our sets, provides a value of 117 ppm, also 2.3 ppm off the experimental value [27]. As no intramolecular H-

bonds exist for this compound in solution, it may be worth adding it in a future training set.

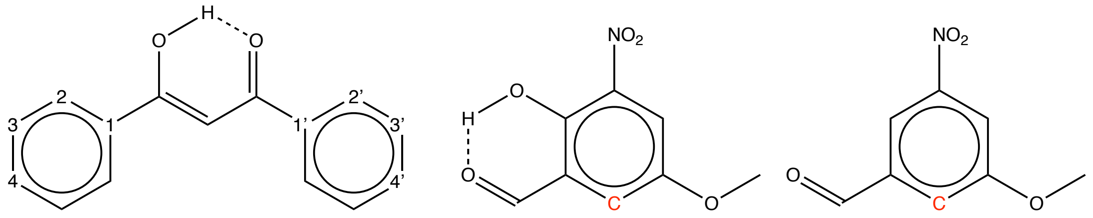

Figure S6. Structures of: major enol form of 1,3-diphenyl-1,3-propanedione with a stabilizing H-bond, 5-methoxy-3-nitrosalicylaldehyde with an intramolecular H-bond and 3-methoxy-5-nitrobenzaldehyde; the carbon atoms mapped for the enol structure, e.g. 1 and 1', are those that are equivalent in the NMR spectrum; the carbons shown in red are those whose chemical shift is difficult to predict.

In view of the preceding discussion (analysis of training set estimations), the case of 5-methoxy-3-nitrosalicylaldehyde would correspond more closely to the fourth case iv, as would that of the blue carbon of 6-bromopiperonal in Figure S5, for which a shift deviation of 3.4 ppm is observed. This is not surprising for the latter, as the training set contains only three molecules with a –C(=O)X-type function having a neighboring bromine atom on the benzene ring (two aldehydes and one ester). This is also why the brominated carbon of *o*-bromobenzaldehyde has a poorly predicted shift (1.7 ppm off). This discrepancy could therefore be reduced by adding a few brominated benzaldehyde derivatives in position 2 in future data sets.

References

1. AIST. Spectral Database for Organic Compounds. Available online: https://sdbs.db.aist.go.jp (accessed on 2024).
2. Haydl, A.M.; Hartwig, J.F. Palladium-Catalyzed Methylation of Aryl, Heteroaryl, and Vinyl Boronate Esters. *Organic Letters* **2019**, *21*, 1337-1341, doi:10.1021/acs.orglett.9b00025.
3. Gómez, J.C.C.; Bernstein, M.; Sýkora, S. An Integrated Approach to Structure Verification Using Automated Procedures. **2015**, 445-492, doi:10.1002/9783527664610.ch12.
4. Cobas, C.; Seoane, F.; Vaz, E.; Bernstein, M.A.; Dominguez, S.; Pérez, M.; Sýkora, S. Automatic assignment of 1H-NMR spectra of small molecules. *Magnetic Resonance in Chemistry* **2013**, *51*, 649-654, doi:10.1002/mrc.3995.
5. Lodewyk, M.W.; Siebert, M.R.; Tantillo, D.J. Computational Prediction of 1H and 13C Chemical Shifts: A Useful Tool for Natural Product, Mechanistic, and Synthetic Organic Chemistry. *Chemical Reviews* **2012**, *112*, 1839-1862, doi:10.1021/cr200106v.
6. Grant, D.M.; Paul, E.G. Carbon-13 Magnetic Resonance. II. Chemical Shift Data for the Alkanes. *Journal of the American Chemical Society* **1964**, *86*, 2984-2990, doi:10.1021/ja01069a004.
7. Lindeman, L.P.; Adama, J.Q. Carbon-13 nuclear magnetic resonance spectrometry. Chemical shifts for the paraffins through C9. *Analytical Chemistry* **1971**, *43*, 1245-1252, doi:10.1021/ac60304a002.
8. Clerc, J.T.; Sommerauer, H. A minicomputer program based on additivity rules for the estimation of 13c-nmr chemical shifts. *Analytica Chimica Acta* **1977**, *95*, 33-40, doi:10.1016/s0003-2670(00)84995-8.
9. Thomas, S.; Brühl, I.; Heilmann, D.; Kleinpeter, E. 13C NMR Chemical Shift Calculations for Some Substituted Pyridines: A Comparative Consideration. *Journal of Chemical Information and Computer Sciences* **1997**, *37*, 726-730, doi:10.1021/ci970440i.
10. Thomas, S.; Stroehl, D.; Kleinpeter, E. Computer Application of an Incremental System for Calculating 13C NMR Spectra of Aromatic Compounds. *Journal of Chemical Information and Computer Sciences* **1994**, *34*, 725-729, doi:10.1021/ci00020a005.
11. Hyodo, K.; Hasegawa, G.; Oishi, N.; Kuroda, K.; Uchida, K. Direct and Catalytic Amide Synthesis from Ketones via Transoximation and Beckmann Rearrangement under Mild Conditions. *The Journal of Organic Chemistry* **2018**, *83*, 13080-13087, doi:10.1021/acs.joc.8b01810.
12. Morisset, E.; Chardon, A.; Rouden, J.; Blanchet, J. Phenysilane and Silicon Tetraacetate: Versatile Promotors for Amide Synthesis. *European Journal of Organic Chemistry* **2020**, *2020*, 388-392, doi:10.1002/ejoc.201901660.
13. Brasche, G.; García-Fortanet, J.; Buchwald, S.L. Twofold C−H Functionalization: Palladium-Catalyzed Ortho Arylation of Anilides. *Organic Letters* **2008**, *10*, 2207-2210, doi:10.1021/ol800619c.
14. Tang, L.; Wang, Z.-L.; Wan, H.-L.; He, Y.-H.; Guan, Z. Visible-Light-Induced Beckmann Rearrangement

by Organic Photoredox Catalysis. *Organic Letters* **2020**, *22*, 6182-6186, doi:10.1021/acs.orglett.0c02168.
15. SciFinder; Chemical Abstracts Service: Columbus, O. Available online: https://scifinder-n.cas.org/ (accessed on 2024).
16. Stuart, J.G.; Khora, S.; McKenney, J.D.; Castle, R.N. The synthesis of dimethoxy- and trimethoxy[1]benzothieno[2,3-c]quinolines. *Journal of Heterocyclic Chemistry* **2009**, *24*, 1589-1594, doi:10.1002/jhet.5570240617.
17. Cakmak, S.; Kutuk, H.; Odabasoglu, M.; Yakan, H.; Buyukgungor, O. Spectroscopic Properties and Preparation of Some 2,3-Dimethoxybenzamide Derivatives. *Letters in Organic Chemistry* **2016**, *13*, 181-194, doi:10.2174/1570178613666151230210931.
18. Hayrapetyan, D.; Rit, R.K.; Kratz, M.; Tschulik, K.; Gooßen, L.J. Electrochemical C−H Cyanation of Electron-Rich (Hetero)Arenes. *Chemistry – A European Journal* **2018**, *24*, 11288-11291, doi:10.1002/chem.201802247.
19. Ewing, D.F. 13C substituent effects in monosubstituted benzenes. *Organic Magnetic Resonance* **1979**, *12*, 499-524, doi:10.1002/mrc.1270120902.
20. Zhan, W.; Ji, L.; Ge, Z.-m.; Wang, X.; Li, R.-t. A continuous-flow synthesis of primary amides from hydrolysis of nitriles using hydrogen peroxide as oxidant. *Tetrahedron* **2018**, *74*, 1527-1532, doi:10.1016/j.tet.2018.02.017.
21. Filleux-Blanchard, M.L.; Fieus, J.; Hallé, J.C. Processus de rotation empéchée autour de la liaison C–N dans les anilines. *Organic Magnetic Resonance* **1973**, *5*, 221-225, doi:10.1002/mrc.1270050506.
22. Van Damme, J.; van den Berg, O.; Brancart, J.; Van Assche, G.; Du Prez, F. A novel donor-π-acceptor anthracene monomer: Towards faster and milder reversible dimerization. *Tetrahedron* **2019**, *75*, 912-920, doi:10.1016/j.tet.2019.01.007.
23. Yong, Q.; Sun, B.; Zhang, F.-L. Palladium-catalyzed ortho-C(sp2) H bromination of benzaldehydes via a monodentate transient directing group strategy. *Tetrahedron Letters* **2019**, *60*, 151263, doi:10.1016/j.tetlet.2019.151263.
24. Hou, J.; Li, Z.; Jia, X.-D.; Liu, Z.-Q. Bromination of Arenes Using I2O5-KBr in Water. *Synthetic Communications* **2013**, *44*, 181-187, doi:10.1080/00397911.2013.796523.
25. Chen, P.; Zhang, Q.-Q.; Guo, J.; Chen, L.-L.; Wang, Y.-B.; Zhang, X. An effective preparation of both 1,3-diketones and nitriles from alkynones with oximes as hydroxide sources. *Organic & Biomolecular Chemistry* **2018**, *16*, 8336-8344, doi:10.1039/c8ob01861h.
26. Baker, R.; Castro, J.L. Total synthesis of (+)-macbecin I. *Journal of the Chemical Society, Perkin Transactions 1* **1990**, 47, doi:10.1039/p19900000047.
27. Brandt, G.E.L.; Blagg, B.S.J. Monoenomycin: A Simplified Trienomycin A Analogue That Manifests Anticancer Activity. *ACS Medicinal Chemistry Letters* **2011**, *2*, 735-740, doi:10.1021/ml200108y.

S3. GRAPH MACHINE DEMONSTRATIONS WITH DOCKER CONTAINERS

Installing Docker for Mac and downloading the demo image

In the following example the installation of Docker is performed with an Intel version of Docker Desktop for Mac. The same operations can be done on a Mac with an Apple ARM-based system (apple silicon). The link to download the appropriate version is given at the end of this Section.

1) Download the Intel chip Docker application by copying and pasting the following line into a browser URL bar:

```
https://desktop.docker.com/mac/main/amd64/Docker.dmg?utm_source=docker&utm_medium=webreferral&utm_campaign=dd-smartbutton&utm_location=module
```

2) After Docker installation (with administrator privileges), launch it. You can open the Docker preferences to change the Docker allocated memory (e.g. 64 Go), and select a number of cores for the virtual machine (more or less according to the machine resources).

3) Open a terminal window, paste the following line, and hit return:

```
docker pull espcigm/metagen-cshift
```

The image used to create containers is then downloaded.

4) You can ensure that the image is genuine by checking the hash code generated at the end of the download process; it should be:

```
sha256: a4053f506924cab23597cc0d2ef6e774f7b11f643232334d1a8e457c587a392c
```

The set up is now complete.

More information on Docker client installation can be obtained from the link below and from the Docker website (docker.com).

http://pubs.acs.org/doi/suppl/10.1021/acs.jcim.0c00083/suppl_file/ci0c00083_si_003.pdf

The link to install the Apple chip version of Docker is the following:

```
https://desktop.docker.com/mac/main/arm64/Docker.dmg?utm_source=docker&utm_medium=w
ebreferral&utm_campaign=dd-smartbutton&utm_location=module
```

Note: to run the graph machine image correctly for this architecture, you need to check the "Use Rosetta for x86_64/amd64 emulation on Apple Silicon" box in the Docker/General settings.

Installing Docker for Windows and downloading the demo image

The steps for the installation of the Docker Windows version and the demo image are given below.

1) Download the Windows version of Docker application by copying and pasting the following line into a browser URL bar:

```
https://desktop.docker.com/win/main/amd64/Docker%20Desktop%20Installer.exe?utm_sour
ce=docker&utm_medium=webreferral&utm_campaign=dd-smartbutton&utm_location=module
```

2) Install the Docker Desktop version 4.30.0 (or above) with administrator privileges
3) In the Dashboard opened at the beginning of the Docker installation verify that:
    a. the box 'Enable Hyper-V Windows feature' is checked;
    b. the box 'Add shortcut to Desktop' is checked;
    c. uncheck if necessary the box 'Install required Windows components for WSL 2'.
4) When the installation has succeeded open Docker Desktop with the desktop shortcut. You have to accept the Docker service agreement by checking the box 'I accept the terms'.
5) Docker Desktop is starting, you might skip the tutorial. Go to Docker Desktop settings and choose General, then uncheck the box 'Use the WSL 2 based engine' (very important). Click on 'Apply and restart'.
6) After a while, open a Powershell window (or a command prompt window) and to verify that Docker is running type the following command:

```
docker version
```

You can confirm in the lines returned that the Docker Desktop version is 4.30.0 (or above).

7) Open a terminal window, paste the following line, and hit return:

```
docker pull espcigm/metagen-cshift
```

The image used to create containers is then downloaded.

8) You can ensure that the image is genuine by checking the hash code generated at the end of the download process; it should be:

```
sha256: a4053f506924cab23597cc0d2ef6e774f7b11f643232334d1a8e457c587a392c
```

The same image is used to launch either graph machine or neural network computations. The set up is now complete.

Notes: 1) If at step 4 Docker does not start, it is because Hyper-V is probably not active on your system. You need to activate Hyper-V by typing in a powershell windows the following command (as admin):

```
Enable-WindowsOptionalFeature -Online -FeatureName Microsoft-Hyper-V -All
```

then restart your machine.

2) Docker Desktop can be installed for a standard user. The user has to be a member of the docker-users group. This can be done with the Windows administration tools.

Installing Docker for Ubuntu 20.04 TLS and downloading the demo image

The steps for the installation of the Docker Ubuntu version and the demo image are given below.

1) Open a terminal window, paste the following line, and hit return:

```
sudo snap install docker
```

2) When the installation is complete, download the docker image with the following command:

```
sudo docker pull espcigm/metagen-cshift
```

The image used to create containers is then downloaded.

Note: sudo is necessary to connect with the docker daemon which is owned by root. If you don't want to have to preface the docker command with sudo, follow the instructions of this page:
https://docs.docker.com/engine/install/linux-postinstall/

Loading and launching the Docker image

To open a container that will launch the default graph machine computations for the molecules of the *test set*, open a terminal window (or start a PowerShell session), and type the following line of text below (or copy and paste it), the argument demo being optional:

```
docker run -it --rm espcigm/metagen-cshift
```

Another computation can be called with the same command line but with the argument demofull. The explanations and the outputs of this command line are given in the Section 'Graph machine results with Docker'.

Notes: 1) No file is created on the host machine; the computed results are lost when the container is deleted.
2) The computed times reported during the demo depend on the machine used.

For more explanations on hyper-V you can use the following link:
https://docs.microsoft.com/fr-fr/virtualization/hyper-v-on-windows/quick-start/enable-hyper-v

Predicting ^{13}C chemical shift for the 156-carbon test set

We explain hereafter the demo that describes the cshift computation for the ten test carbons with the

selected complexity of the graph machine-based model; the command line for launching this demo is the following:

```
docker run -it --rm espcigm/metagen-cshift
```

The data file used for the computation of the `cshift` values of the test set molecules is the file Test156.xlsx, that can be downloaded separately. The file sheet contains the data for the 156 carbons of the test set. Every line of this sheet contains at least a carbon name, which derives from the name of the molecule, its specific SMILES code for the atom in question, and the experimental value of the property of interest (here the carbon chemical shift, cshift).

During the training step *achieved in an earlier stage*, a graph machine model based on the SMILES code derived from the molecular structure was automatically generated for every benzenic carbon of the training set. All 10577 models were then merged into a module that was trained with the desired property values. The parameters at the end of the training were stored to be used with a new model.

In the course of the present demo a graph machine model is generated for the 10 first carbons (also inputted as their SMILES codes) of the 10 first lines of the sheet. After the model constructions, the parameters saved during the training step are passed to the functions of the 10 graph machines to predict the cshift values of the test set carbons.

Explanation of the demo command line

The execution of the graph machine demonstration can be launched from the command line. The proposed default command line is (demo can be omitted):

```
docker run -it --rm espcigm/metagen-cshift demo
```

It contains the following terms:

- "docker": calls the Docker daemon of the host machine;
- "run": launches a Docker container from the Docker image;
- "-it": opens and launches the interactive mode;
- "--rm": destroys the container at the end of the session;
- "espcigm/metagen-cshift": name of the Docker image launched with the run command;
- "demo": this argument invokes the graph machines mode and a SMILES input is expected. The demo computations are made with the graph machines model that has the number of hidden neurons chosen when looking for the appropriate complexity, i.e. 26 hidden neurons. After completion of the demo, the container is automatically deleted. A new demo session can be started with the same command, but within a new container. If "demofull" is used as argument, instead of "demo", the cshift predictions are computed for the 156 carbons of the test set. A progress indicator is then displayed, providing information on the duration of the computation. Be careful, the calculation can take some time!

Other command line option

One subcommand 'get' can be appended to the command line instead of using demo or demofull. This subcommand, when passed to the demonstrator, allows the computation of the property value for a single compound using a SMILES code input as follows:

```
docker run -it --rm espcigm/metagen-cshift get "id;input"
```

where "id" is the name of the carbon and "input" corresponds to a SMILES code. When the computation is done, the results are written in the terminal window. Some examples are given in the next Section, paragraph 'Prediction of cshift for a single carbon of the test set'.

The use of Docker for graph machines was described in greater detail in previous publications, for example in the file available at:

https://pubs.acs.org/doi/suppl/10.1021/acs.jcim.7b00512/suppl_file/ci7b00512_si_001.pdf.

S4. GRAPH MACHINE RESULTS WITH DOCKER

The following computations were made on a M2 ultra 3.6 GHz 24-Core Apple silicon with 192 GB of RAM running macOs Sonoma 14 (Docker configuration: 24 CPUs, 48 GB allocated RAM, 1 Go swap). All graph machine tasks were launched with the maximum number of available CPUs minus 2, i.e. 22 CPUs on the M2.

Predictions of the cshift values for the first 10-carbon test set

For the graph machine model with 26 hidden neurons, the predictions for the first ten carbons are obtained with the following command (the final demo can be omitted):

```
docker run -it --rm espcigm/metagen-cshift demo
```

The terminal output is then:

```
Running demo
computing time: 20.64 s
results:
```

C-NAMES	SMILES	Target	CSHIFT	Estimated_CSHIFT	MIN_CSHIFT	MAX_CSHIFT
5-bromo-1,2,3-trimethoxybenzene0	CO[c:1]1cc(Br)cc(OC)c1OC	C3	154.0	154.1	153.2	155.1
5-bromo-1,2,3-trimethoxybenzene1	COc1[c:1]c(Br)cc(OC)c1OC	C4	109.1	109.8	108.7	111.0
5-bromo-1,2,3-trimethoxybenzene2	COc1c[c:1](Br)cc(OC)c1OC	C5	116.2	113.3	111.6	115.2
5-bromo-1,2,3-trimethoxybenzene3	COc1cc(Br)cc(OC)[c:1]1OC	C11	137.5	137.9	137.0	139.1
2-bromo-4-methoxy-6-nitrophenol0	O[c:1]1c([N+]([O-])=O)cc(OC)cc1Br	C2	147.1	145.8	143.0	148.0
2-bromo-4-methoxy-6-nitrophenol1	Oc1[c:1]([N+]([O-])=O)cc(OC)cc1Br	C3	133.7	134.0	131.9	135.5
2-bromo-4-methoxy-6-nitrophenol2	Oc1c([N+]([O-])=O)[c:1]c(OC)cc1Br	C7	106.4	109.0	107.6	110.4
2-bromo-4-methoxy-6-nitrophenol3	Oc1c([N+]([O-])=O)c[c:1](OC)cc1Br	C8	152.2	152.9	151.6	153.7
2-bromo-4-methoxy-6-nitrophenol4	Oc1c([N+]([O-])=O)cc(OC)[c:1]c1Br	C11	129.8	124.8	123.4	126.5
2-bromo-4-methoxy-6-nitrophenol5	Oc1c([N+]([O-])=O)cc(OC)c[c:1]1Br	C12	113.8	113.9	112.1	115.3

```
done
```

For the 10 above atoms, the cshift predictions are the same as those reported as prediction values in column E of the Table S9 (excel file CS10577A-156T-1011T-SI) for the first 10 test carbons (entries 1−10).

Explanation: the first carbon listed, whose name is 5-bromo-1,2,3-trimethoxybenzene0, belongs to the molecule 5-bromo-1,2,3-trimethoxybenzene shown on the left in the following figure. On the right, the atoms have been numbered according to the SMILES carbon code (the first atom in the code is number 1). The carbon of interest is in third position, indicated in red, and it has a predicted shift value of 154.1 ppm. As the molecule is symmetrical, the shifts of carbons 4 and 7 as well as carbons 3 and 8 are equal. That is why only cshifts for targets C3, C4, C5 and C11 are computed. For a molecule without symmetry, like 2-bromo-4-methoxy-6-nitrophenol, which is second on the list, the shifts of all six benzenic carbons are computed with the GM26 model.

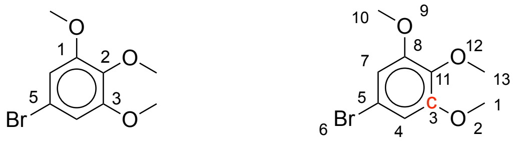

5-bromo-1,2,3-trimethoxybenzene 5-bromo-1,2,3-trimethoxybenzene0
COc1cc(Br)cc(OC)c1OC CO[c:1]1cc(Br)cc(OC)c1OC

Structure visualization with carbon number: if you do not have a molecule editor, you can install version 1.99 of Avogadro 2, an open-source program available at https://two.avogadro.cc/install/index.html. Once installed, go to the construction menu, submenu insert and code SMILES. Paste the code "CO[c:1]1cc(Br)cc(OC)c1OC" directly from the first carbon in the list (5-bromo-1,2,3-trimethoxybenzene0) and hit return. The molecule is then displayed in 3D view. In the left-hand side panel, click the “labels” box, then in the "View configurations" panel, choose the index option for the nature of the atom labels. Carbon 3 is easily identified and corresponds to the atom shown in the 2D structure on the right above.

Estimations of the cshift values for the 156-carbon test set

For the graph machine model with 26 hidden neurons, the estimations for the 26 molecules of the test set

are obtained with the following command:

```
docker run -it --rm espcigm/metagen-cshift demofull
```

The terminal output is then:

```
Run demo full test
computing: 100%|██████████████████████████████| 156/156 [04:22<00:00, 1.68s/it]
results:
```

C-NAMES	SMILES	Target	CSHIFT	Estimated_CSHIFT	MIN_CSHIFT	MAX_CSHIFT
5-bromo-1,2,3-trimethoxybenzene0	CO[c:1]1cc(Br)cc(OC)c1OC	C3	154.0	154.1	153.2	155.1
5-bromo-1,2,3-trimethoxybenzene1	COc1[c:1]c(Br)cc(OC)c1OC	C4	109.1	109.8	108.7	111.0
5-bromo-1,2,3-trimethoxybenzene2	COc1c[c:1](Br)cc(OC)c1OC	C5	116.2	113.3	111.6	115.2
5-bromo-1,2,3-trimethoxybenzene3	COc1cc(Br)cc(OC)[c:1]1OC	C11	137.5	137.9	137.0	139.1
2-bromo-4-methoxy-6-nitrophenol0	O[c:1]1c([N+]([O-])=O)cc(OC)cc1Br	C2	147.1	145.8	143.0	148.0
2-bromo-4-methoxy-6-nitrophenol1	Oc1[c:1]([N+]([O-])=O)cc(OC)cc1Br	C3	133.7	134.0	131.9	135.5
2-bromo-4-methoxy-6-nitrophenol2	Oc1c([N+]([O-])=O)[c:1]c(OC)cc1Br	C7	106.4	109.0	107.6	110.4
2-bromo-4-methoxy-6-nitrophenol3	Oc1c([N+]([O-])=O)c[c:1](OC)cc1Br	C8	152.2	152.9	151.6	153.7
2-bromo-4-methoxy-6-nitrophenol4	Oc1c([N+]([O-])=O)cc(OC)[c:1]c1Br	C11	129.8	124.8	123.4	126.5
2-bromo-4-methoxy-6-nitrophenol5	Oc1c([N+]([O-])=O)cc(OC)c[c:1]1Br	C12	113.8	113.9	112.1	115.3
1-benzyloxy-4-iodobenzene0	I[c:1]1ccc(OCc2ccccc2)cc1	C2	83.1	83.3	81.0	85.2
1-benzyloxy-4-iodobenzene1	Ic1[c:1]cc(OCc2ccccc2)cc1	C3	138.3	138.4	136.7	139.8
1-benzyloxy-4-iodobenzene2	Ic1c[c:1]c(OCc2ccccc2)cc1	C4	117.3	116.6	114.1	118.7
1-benzyloxy-4-iodobenzene3	Ic1cc[c:1](OCc2ccccc2)cc1	C5	158.7	158.2	156.3	159.7
1-benzyloxy-4-iodobenzene4	Ic1ccc(OC[c:1]2ccccc2)cc1	C8	136.5	138.2	135.8	141.5
1-benzyloxy-4-iodobenzene5	Ic1ccc(OCc2[c:1]cccc2)cc1	C9	127.4	128.2	126.7	129.2
1-benzyloxy-4-iodobenzene6	Ic1ccc(OCc2c[c:1]ccc2)cc1	C10	127.6	128.6	127.2	129.9
1-benzyloxy-4-iodobenzene7	Ic1ccc(OCc2cc[c:1]cc2)cc1	C11	128.1	127.9	127.0	129.1
4-bromo-3-nitrobenzaldehyde0	O=C[c:1]1ccc(Br)c([N+]([O-])=O)c1	C3	136.1	136.3	134.5	137.6
4-bromo-3-nitrobenzaldehyde1	O=Cc1[c:1]cc(Br)c([N+]([O-])=O)c1	C4	132.6	134.9	133.8	136.4
4-bromo-3-nitrobenzaldehyde2	O=Cc1c[c:1]c(Br)c([N+]([O-])=O)c1	C5	136.3	136.2	135.5	137.1
4-bromo-3-nitrobenzaldehyde3	O=Cc1cc[c:1](Br)c([N+]([O-])=O)c1	C6	121.1	121.4	119.1	122.9
4-bromo-3-nitrobenzaldehyde4	O=Cc1ccc(Br)[c:1]([N+]([O-])=O)c1	C8	150.5	149.8	148.4	151.1
4-bromo-3-nitrobenzaldehyde5	O=Cc1ccc(Br)c([N+]([O-])=O)[c:1]1	C12	126.1	126.6	125.0	127.9
3-bromo-4-methylbenzaldehyde0	O=C[c:1]1ccc(C)c(Br)c1	C3	136.0	135.3	134.4	136.3
3-bromo-4-methylbenzaldehyde1	O=Cc1[c:1]cc(C)c(Br)c1	C4	128.4	128.5	127.8	129.3

3-bromo-4-methylbenzaldehyde2	O=Cc1c[c:1]c(C)c(Br)c1	C5	131.4	131.3	130.4	132.4
3-bromo-4-methylbenzaldehyde3	O=Cc1cc[c:1](C)c(Br)c1	C6	145.2	145.3	144.1	146.8
3-bromo-4-methylbenzaldehyde4	O=Cc1ccc(C)[c:1](Br)c1	C8	125.7	125.3	124.1	126.4
3-bromo-4-methylbenzaldehyde5	O=Cc1ccc(C)c(Br)[c:1]1	C10	133.5	133.1	132.0	134.2
3,5-dibromobenzyl alcohol0	Br[c:1]1cc(CO)cc(Br)c1	C2	123.1	123.1	122.4	123.6
3,5-dibromobenzyl alcohol1	Brc1[c:1]c(CO)cc(Br)c1	C3	128.4	128.7	128.1	129.4
3,5-dibromobenzyl alcohol2	Brc1c[c:1](CO)cc(Br)c1	C4	144.7	144.3	143.5	145.1
3,5-dibromobenzyl alcohol3	Brc1cc(CO)cc(Br)[c:1]1	C10	133.1	133.3	132.5	133.9
ethyl p-bromophenylacetate0	O=C(OCC)C[c:1]1ccc(Br)cc1	C7	133.1	131.8	126.4	135.4
ethyl p-bromophenylacetate1	O=C(OCC)Cc1[c:1]cc(Br)cc1	C8	131.0	129.6	126.5	131.4
ethyl p-bromophenylacetate2	O=C(OCC)Cc1c[c:1]c(Br)cc1	C9	131.7	131.4	130.1	132.7
ethyl p-bromophenylacetate3	O=C(OCC)Cc1cc[c:1](Br)cc1	C10	121.1	121.2	116.9	123.4
2-iodo-4-methylaniline0	N[c:1]1ccc(C)cc1I	C2	144.3	144.4	143.0	146.2
2-iodo-4-methylaniline1	Nc1[c:1]cc(C)cc1I	C3	114.7	113.9	112.2	115.5
2-iodo-4-methylaniline2	Nc1c[c:1]c(C)cc1I	C4	130.1	129.8	129.1	130.7
2-iodo-4-methylaniline3	Nc1cc[c:1](C)cc1I	C5	129.6	128.6	127.0	130.1
2-iodo-4-methylaniline4	Nc1ccc(C)[c:1]c1I	C7	139.0	138.7	137.2	140.4
2-iodo-4-methylaniline5	Nc1ccc(C)c[c:1]1I	C8	84.4	84.1	82.5	85.6
p-(trifluoromethylthio)bromobenzene0	FC(F)(F)S[c:1]1ccc(Br)cc1	C6	123.5	125.6	116.4	137.7
p-(trifluoromethylthio)bromobenzene1	FC(F)(F)Sc1[c:1]cc(Br)cc1	C7	137.7	133.9	128.3	140.2
p-(trifluoromethylthio)bromobenzene2	FC(F)(F)Sc1c[c:1]c(Br)cc1	C8	132.8	132.6	129.7	136.5
p-(trifluoromethylthio)bromobenzene3	FC(F)(F)Sc1cc[c:1](Br)cc1	C9	126.0	124.1	119.3	127.8
methyl 2-amino-4-(trifluoromethyl)benzoate0	O=C(OC)[c:1]1ccc(C(F)(F)F)cc1N	C5	113.2	114.6	112.4	116.2
methyl 2-amino-4-(trifluoromethyl)benzoate1	O=C(OC)c1[c:1]cc(C(F)(F)F)cc1N	C6	132.2	131.6	130.2	132.6
methyl 2-amino-4-(trifluoromethyl)benzoate2	O=C(OC)c1c[c:1]c(C(F)(F)F)cc1N	C7	112.4	113.5	111.8	114.6
methyl 2-amino-4-(trifluoromethyl)benzoate3	O=C(OC)c1cc[c:1](C(F)(F)F)cc1N	C8	135.5	136.9	135.5	138.6
methyl 2-amino-4-(trifluoromethyl)benzoate4	O=C(OC)c1ccc(C(F)(F)F)[c:1]c1N	C13	113.5	113.5	112.2	114.7
methyl 2-amino-4-(trifluoromethyl)benzoate5	O=C(OC)c1ccc(C(F)(F)F)c[c:1]1N	C14	150.2	150.9	149.6	152.5
methyl 5-bromo-2-methyl-3-nitrobenzoate0	O=C(OC)[c:1]1cc(Br)cc([N+]([O-])=O)c1C	C5	134.7	135.5	133.5	137.1

methyl 5-bromo-2-methyl-3-nitrobenzoate1	O=C(OC)c1[c:1]c(Br)cc([N+]([O-])=O)c1C	C6	136.5	135.2	133.9	136.5
methyl 5-bromo-2-methyl-3-nitrobenzoate2	O=C(OC)c1c[c:1](Br)cc([N+]([O-])=O)c1C	C7	119.2	119.9	117.8	121.4
methyl 5-bromo-2-methyl-3-nitrobenzoate3	O=C(OC)c1cc(Br)[c:1]c([N+]([O-])=O)c1C	C9	129.5	129.3	128.0	130.9
methyl 5-bromo-2-methyl-3-nitrobenzoate4	O=C(OC)c1cc(Br)c[c:1]([N+]([O-])=O)c1C	C10	152.4	151.2	149.7	153.3
methyl 5-bromo-2-methyl-3-nitrobenzoate5	O=C(OC)c1cc(Br)cc([N+]([O-])=O)[c:1]1C	C14	132.1	132.4	129.0	135.2
methyl 4-hydroxy-3,5-dimethoxybenzoate0	O=C(OC)[c:1]1cc(OC)c(O)c(OC)c1	C5	121.2	122.7	120.8	124.3
methyl 4-hydroxy-3,5-dimethoxybenzoate1	O=C(OC)c1[c:1]c(OC)c(O)c(OC)c1	C6	106.8	105.4	103.2	106.8
methyl 4-hydroxy-3,5-dimethoxybenzoate2	O=C(OC)c1c[c:1](OC)c(O)c(OC)c1	C7	146.7	146.4	145.4	147.3
methyl 4-hydroxy-3,5-dimethoxybenzoate3	O=C(OC)c1cc(OC)[c:1](O)c(OC)c1	C10	139.3	140.0	139.0	141.1
4-chloro-1-iodo-2-nitrobenzene0	O=[N+]([c:1]1cc(Cl)ccc1I)[O-]	C3	153.5	152.9	151.2	154.5
4-chloro-1-iodo-2-nitrobenzene1	O=[N+](c1[c:1]c(Cl)ccc1I)[O-]	C4	125.7	124.9	124.0	126.1
4-chloro-1-iodo-2-nitrobenzene2	O=[N+](c1c[c:1](Cl)ccc1I)[O-]	C5	135.4	135.0	134.0	136.4
4-chloro-1-iodo-2-nitrobenzene3	O=[N+](c1cc(Cl)[c:1]cc1I)[O-]	C7	133.6	135.4	134.8	136.2
4-chloro-1-iodo-2-nitrobenzene4	O=[N+](c1cc(Cl)c[c:1]c1I)[O-]	C8	142.8	142.4	141.3	144.0
4-chloro-1-iodo-2-nitrobenzene5	O=[N+](c1cc(Cl)cc[c:1]1I)[O-]	C9	83.5	84.0	82.3	86.5
4-bromo-2-methylbenzyl alcohol0	C[c:1]1cc(Br)ccc1CO	C2	138.3	137.3	136.4	138.9
4-bromo-2-methylbenzyl alcohol1	Cc1[c:1]c(Br)ccc1CO	C3	133.1	133.3	132.5	134.2
4-bromo-2-methylbenzyl alcohol2	Cc1c[c:1](Br)ccc1CO	C4	121.5	120.8	119.8	121.5
4-bromo-2-methylbenzyl alcohol3	Cc1cc(Br)[c:1]cc1CO	C6	129.0	129.0	128.5	129.7
4-bromo-2-methylbenzyl alcohol4	Cc1cc(Br)c[c:1]c1CO	C7	129.1	129.1	128.3	130.3
4-bromo-2-methylbenzyl alcohol5	Cc1cc(Br)cc[c:1]1CO	C8	137.7	137.8	136.5	138.7
isoeugenyl methyl ether0	CO[c:1]1ccc(/C=C/C)cc1OC	C3	148.3	148.7	147.7	150.1
isoeugenyl methyl ether1	COc1[c:1]cc(/C=C/C)cc1OC	C4	111.3	112.1	110.2	114.6
isoeugenyl methyl ether2	COc1c[c:1]c(/C=C/C)cc1OC	C5	118.7	119.5	117.5	121.4
isoeugenyl methyl ether3	COc1cc[c:1](/C=C/C)cc1OC	C6	131.2	132.2	129.8	134.5
isoeugenyl methyl ether4	COc1ccc(/C=C/C)[c:1]c1OC	C10	108.6	109.9	107.1	112.6
isoeugenyl methyl ether5	COc1ccc(/C=C/C)c[c:1]1OC	C11	149.1	149.8	148.5	150.9
2-bromoresorcinol0	O[c:1]1cccc(O)c1Br	C2	153.0	153.7	152.1	155.5
2-bromoresorcinol1	Oc1[c:1]ccc(O)c1Br	C3	108.2	108.1	107.2	109.3

2-bromoresorcinol2	Oc1c[c:1]cc(O)c1Br	C4	129.1	129.8	128.9	130.5
2-bromoresorcinol3	Oc1cccc(O)[c:1]1Br	C8	99.4	97.9	94.5	100.8
2-ethoxy-5-(1-propenyl)phenol0	O[c:1]1cc(/C=C/C)ccc1OCC	C2	145.9	146.1	144.3	147.5
2-ethoxy-5-(1-propenyl)phenol1	Oc1[c:1]c(/C=C/C)ccc1OCC	C3	111.5	113.0	110.0	115.7
2-ethoxy-5-(1-propenyl)phenol2	Oc1c[c:1](/C=C/C)ccc1OCC	C4	131.8	132.2	129.7	134.0
2-ethoxy-5-(1-propenyl)phenol3	Oc1cc(/C=C/C)[c:1]cc1OCC	C8	118.1	119.7	118.1	121.3
2-ethoxy-5-(1-propenyl)phenol4	Oc1cc(/C=C/C)c[c:1]c1OCC	C9	111.6	112.4	110.7	114.4
2-ethoxy-5-(1-propenyl)phenol5	Oc1cc(/C=C/C)cc[c:1]1OCC	C10	145.0	145.8	144.7	147.2
2,5-difluoro-4-nitrotoluene0	C[c:1]1cc(F)c([N+]([O-])=O)cc1F	C2	134.8	134.4	132.7	136.1
2,5-difluoro-4-nitrotoluene1	Cc1[c:1]c(F)c([N+]([O-])=O)cc1F	C3	120.5	120.9	119.1	122.1
2,5-difluoro-4-nitrotoluene2	Cc1c[c:1](F)c([N+]([O-])=O)cc1F	C4	151.7	152.6	151.6	153.8
2,5-difluoro-4-nitrotoluene3	Cc1cc(F)[c:1]([N+]([O-])=O)cc1F	C6	135.1	135.5	134.6	136.6
2,5-difluoro-4-nitrotoluene4	Cc1cc(F)c([N+]([O-])=O)[c:1]c1F	C10	112.6	112.2	111.2	113.7
2,5-difluoro-4-nitrotoluene5	Cc1cc(F)c([N+]([O-])=O)c[c:1]1F	C11	155.9	156.4	155.5	157.4
2,4-dichlorobenzonitrile0	N#C[c:1]1ccc(Cl)cc1Cl	C3	112.0	110.8	109.6	111.8
2,4-dichlorobenzonitrile1	N#Cc1[c:1]cc(Cl)cc1Cl	C4	134.6	134.6	133.7	135.5
2,4-dichlorobenzonitrile2	N#Cc1c[c:1]c(Cl)cc1Cl	C5	127.9	127.9	127.2	128.9
2,4-dichlorobenzonitrile3	N#Cc1cc[c:1](Cl)cc1Cl	C6	140.1	139.5	138.7	140.3
2,4-dichlorobenzonitrile4	N#Cc1ccc(Cl)[c:1]c1Cl	C8	130.3	130.5	129.8	131.3
2,4-dichlorobenzonitrile5	N#Cc1ccc(Cl)c[c:1]1Cl	C9	137.9	137.8	136.5	138.8
2,5-dichlorobenzonitrile0	N#C[c:1]1cc(Cl)ccc1Cl	C3	114.8	113.6	112.7	114.6
2,5-dichlorobenzonitrile1	N#Cc1[c:1]c(Cl)ccc1Cl	C4	133.5	133.4	132.7	134.1
2,5-dichlorobenzonitrile2	N#Cc1c[c:1](Cl)ccc1Cl	C5	133.3	133.1	132.3	133.7
2,5-dichlorobenzonitrile3	N#Cc1cc(Cl)[c:1]cc1Cl	C7	134.1	134.0	133.4	134.5
2,5-dichlorobenzonitrile4	N#Cc1cc(Cl)c[c:1]c1Cl	C8	131.2	131.3	130.5	132.0
2,5-dichlorobenzonitrile5	N#Cc1cc(Cl)cc[c:1]1Cl	C9	135.4	135.3	134.6	136.1
methyl 2,3-diaminobenzoate0	O=C(OC)[c:1]1cccc(N)c1N	C5	112.1	111.9	108.6	116.8
methyl 2,3-diaminobenzoate1	O=C(OC)c1[c:1]ccc(N)c1N	C6	122.7	119.9	117.6	122.4
methyl 2,3-diaminobenzoate2	O=C(OC)c1c[c:1]cc(N)c1N	C7	116.7	116.4	115.3	117.7

methyl 2,3-diaminobenzoate3	O=C(OC)c1cc[c:1]c(N)c1N	C8	120.7	120.0	118.2	122.0
methyl 2,3-diaminobenzoate4	O=C(OC)c1ccc[c:1](N)c1N	C9	134.3	135.2	133.2	138.0
methyl 2,3-diaminobenzoate5	O=C(OC)c1cccc(N)[c:1]1N	C11	141.2	139.2	135.8	142.3
2,3,5,6-tetrafluorobenzonitrile0	N#C[c:1]1c(F)c(F)cc(F)c1F	C3	95.4	95.9	94.0	98.6
2,3,5,6-tetrafluorobenzonitrile1	N#Cc1[c:1](F)c(F)cc(F)c1F	C4	147.3	147.4	145.7	149.0
2,3,5,6-tetrafluorobenzonitrile2	N#Cc1c(F)[c:1](F)cc(F)c1F	C6	147.2	147.0	146.0	148.2
2,3,5,6-tetrafluorobenzonitrile3	N#Cc1c(F)c(F)[c:1]c(F)c1F	C8	112.1	111.7	110.5	113.7
4,4',-dimethylbenzhydrol0	OC([c:1]1ccc(C)cc1)c2ccc(C)cc2	C3	141.2	141.3	136.3	143.9
4,4',-dimethylbenzhydrol1	OC(c1[c:1]cc(C)cc1)c2ccc(C)cc2	C4	126.5	126.7	124.0	129.6
4,4',-dimethylbenzhydrol2	OC(c1c[c:1]c(C)cc1)c2ccc(C)cc2	C5	129.1	128.9	127.4	130.6
4,4',-dimethylbenzhydrol3	OC(c1cc[c:1](C)cc1)c2ccc(C)cc2	C6	137.2	137.2	135.6	139.0
4,4',-dipivaloyloxybenzophenone0	O=C([c:1]1ccc(OC(C(C)(C)C)=O)cc1)c2ccc(OC(C(C)(C)C)=O)cc2	C3	134.9	134.6	129.5	136.8
4,4',-dipivaloyloxybenzophenone1	O=C(c1[c:1]cc(OC(C(C)(C)C)=O)cc1)c2ccc(OC(C(C)(C)C)=O)cc2	C4	131.6	131.0	128.7	133.6
4,4',-dipivaloyloxybenzophenone2	O=C(c1c[c:1]c(OC(C(C)(C)C)=O)cc1)c2ccc(OC(C(C)(C)C)=O)cc2	C5	121.6	120.0	113.8	123.1
4,4',-dipivaloyloxybenzophenone3	O=C(c1cc[c:1](OC(C(C)(C)C)=O)cc1)c2ccc(OC(C(C)(C)C)=O)cc2	C6	154.5	154.1	144.7	162.8
diphenyl terephthalate0	O=C(O[c:1]1ccccc1)c2ccc(C(Oc3ccccc3)=O)cc2	C4	150.8	150.2	147.3	152.7
diphenyl terephthalate1	O=C(Oc1[c:1]cccc1)c2ccc(C(Oc3ccccc3)=O)cc2	C5	121.6	122.0	119.8	123.7
diphenyl terephthalate2	O=C(Oc1c[c:1]ccc1)c2ccc(C(Oc3ccccc3)=O)cc2	C6	129.6	129.7	128.8	130.6
diphenyl terephthalate3	O=C(Oc1cc[c:1]cc1)c2ccc(C(Oc3ccccc3)=O)cc2	C7	126.2	126.3	125.0	128.3
diphenyl terephthalate4	O=C(Oc1ccccc1)[c:1]2ccc(C(Oc3ccccc3)=O)cc2	C10	134.0	134.6	131.6	137.3
diphenyl terephthalate5	O=C(Oc1ccccc1)c2[c:1]cc(C(Oc3ccccc3)=O)cc2	C11	130.3	130.3	128.8	132.0
benzyl 4-chlorophenyl ketone0	O=C(C[c:1]1ccccc1)c2ccc(Cl)cc2	C4	134.2	132.3	128.6	138.0
benzyl 4-chlorophenyl ketone1	O=C(Cc1[c:1]cccc1)c2ccc(Cl)cc2	C5	129.4	129.0	125.7	134.9
benzyl 4-chlorophenyl ketone2	O=C(Cc1c[c:1]ccc1)c2ccc(Cl)cc2	C6	128.8	128.1	126.7	130.1
benzyl 4-chlorophenyl ketone3	O=C(Cc1cc[c:1]cc1)c2ccc(Cl)cc2	C7	127.1	127.3	123.8	129.0
benzyl 4-chlorophenyl ketone4	O=C(Cc1ccccc1)[c:1]2ccc(Cl)cc2	C10	134.9	135.6	133.3	137.9
benzyl 4-chlorophenyl ketone5	O=C(Cc1ccccc1)c2[c:1]cc(Cl)cc2	C11	130.1	128.7	126.5	130.6
benzyl 4-chlorophenyl ketone6	O=C(Cc1ccccc1)c2c[c:1]c(Cl)cc2	C12	129.0	129.1	127.5	130.4
benzyl 4-chlorophenyl ketone7	O=C(Cc1ccccc1)c2cc[c:1](Cl)cc2	C13	139.6	139.3	138.4	140.2

4,4,4-trifluoro-1-(4-methoxyphenyl)-1,3-butanedione0	O=C([c:1]1ccc(OC)cc1)CC(C(F)(F)F)=O	C3	125.5	128.9	123.4	136.0
4,4,4-trifluoro-1-(4-methoxyphenyl)-1,3-butanedione1	O=C(c1[c:1]cc(OC)cc1)CC(C(F)(F)F)=O	C4	130.1	130.3	124.8	132.5
4,4,4-trifluoro-1-(4-methoxyphenyl)-1,3-butanedione2	O=C(c1c[c:1]c(OC)cc1)CC(C(F)(F)F)=O	C5	114.5	113.7	112.0	114.6
4,4,4-trifluoro-1-(4-methoxyphenyl)-1,3-butanedione3	O=C(c1cc[c:1](OC)cc1)CC(C(F)(F)F)=O	C6	164.7	163.6	162.0	164.6
4-benzyloxy-3-methoxyphenylacetic acid0	O=C(O)C[c:1]1ccc(OCc2ccccc2)c(OC)c1	C5	126.3	126.8	125.0	128.9
4-benzyloxy-3-methoxyphenylacetic acid1	O=C(O)Cc1[c:1]cc(OCc2ccccc2)c(OC)c1	C6	121.6	122.5	120.3	124.1
4-benzyloxy-3-methoxyphenylacetic acid2	O=C(O)Cc1c[c:1]c(OCc2ccccc2)c(OC)c1	C7	114.2	113.2	110.6	116.0
4-benzyloxy-3-methoxyphenylacetic acid3	O=C(O)Cc1cc[c:1](OCc2ccccc2)c(OC)c1	C8	147.6	147.4	143.9	149.7
4-benzyloxy-3-methoxyphenylacetic acid4	O=C(O)Cc1ccc(OCc2ccccc2)[c:1](OC)c1	C17	149.8	150.0	148.2	152.5
4-benzyloxy-3-methoxyphenylacetic acid5	O=C(O)Cc1ccc(OCc2ccccc2)c(OC)[c:1]1	C20	113.2	112.5	110.1	114.4
4-benzyloxy-3-methoxyphenylacetic acid6	O=C(O)Cc1ccc(OC[c:1]2ccccc2)c(OC)c1	C11	137.2	137.5	133.7	141.9
4-benzyloxy-3-methoxyphenylacetic acid7	O=C(O)Cc1ccc(OCc2[c:1]cccc2)c(OC)c1	C12	127.3	128.1	126.8	129.4
4-benzyloxy-3-methoxyphenylacetic acid8	O=C(O)Cc1ccc(OCc2c[c:1]ccc2)c(OC)c1	C13	128.6	128.4	126.9	129.8
4-benzyloxy-3-methoxyphenylacetic acid9	O=C(O)Cc1ccc(OCc2cc[c:1]cc2)c(OC)c1	C14	127.8	127.9	127.0	129.2

done

For the 156 above atoms, the cshift predictions are the same as those reported as prediction values in column E of Table S9 of excel file CS10577A-156T-1011T-SI.xlsx (entries 1–156).

Prediction of cshift for a single carbon of the test set

Cshift prediction of a given test carbon can be replicated for the default graph-machine-based model. The functional carbon of 2,3,5,6-tetrafluorobenzonitrile (cCN) is given as an example. According to Table S9 in the CS10577A-156T-1011T-SI.xlsx file, the following information can be extracted (entry 117) for this carbon:

Carbon name	SMILES code	$CSHIFT_{exp}$
2,3,5,6-tetrafluorobenzonitrile0	N#C[c:1]1c(F)c(F)cc(F)c1F	95.4

The command used to predict its cshift from SMILES is the following:

```
docker run -it --rm espcigm/metagen-cshift get "2,3,5,6-tetrafluorobenzonitrile0;N#C[c:1]1c(F)c(F)cc(F)c1F"
```

The output produced is then:

```
Running get 2,3,5,6-tetrafluorobenzonitrile0;N#C[c:1]1c(F)c(F)cc(F)c1F, mode GM
computing time: 9.68 s
results:
+----------------------------------+---------------------------+----------+--------------------+--------------+--------------+
| ID                               | smiles                    | Target   |   Estimated_CSHIFT |   MIN_CSHIFT |   MAX_CSHIFT |
|----------------------------------+---------------------------+----------+--------------------+--------------+--------------|
| 2,3,5,6-tetrafluorobenzonitrile0 | N#C[c:1]1c(F)c(F)cc(F)c1F | C3       |               95.9 |         93.4 |         97.9 |
+----------------------------------+---------------------------+----------+--------------------+--------------+--------------+
do
```

The results are identical to those computed for 2,3,5,6-tetrafluorobenzonitrile0 in column E of Table S9.

Prediction of cshift for other carbons

Cshift prediction for any benzenic carbon of a molecule containing atoms among the eleven atoms present in the training data set, namely carbon, hydrogen, oxygen, nitrogen, halogens, sulfur, phosphorus and silicon, can also be computed with a similar command line starting from its SMILES code.

The carbon carrying the chlorine atom in (4-chlorophenyl)trimethylsilane that belongs to the 1011-carbon test set is used as an example. According to Table S10 in the CS10577A-156T-1011T-SI.xlsx file, the following information can be extracted (entry 911):

Carbon name	SMILES code	$CSHIFT_{exp}$
(4-chlorophenyl)trimethylsilane0	C[Si](C)(C)c1cc[c:1](Cl)cc1	135.2

The command below is then entered in the terminal window to predict the cshift value of the chlorine-carrying carbon in (4-chlorophenyl)trimethylsilane from the SMILES input:

```
docker run -it --rm espcigm/metagen-cshift get "(4-chlorophenyl)trimethylsilane0;C[Si](C)(C)c1cc[c:1](Cl)cc1"
```

The returned messages are the following:

```
computing time: 10.44 s
results:
+---------------------------------+-----------------------------+----------+--------------------+--------------+--------------+
| ID                              | smiles                      | Target   |   Estimated_CSHIFT |   MIN_CSHIFT |   MAX_CSHIFT |
|---------------------------------+-----------------------------+----------+--------------------+--------------+--------------|
| 4-chlorophenyl)trimethylsilane0 | C[Si](C)(C)c1cc[c:1](Cl)cc1 | C8       |              135.0 |        133.5 |        135.9 |
+---------------------------------+-----------------------------+----------+--------------------+--------------+--------------+
done
```

The cshift prediction is equal to 135.0 ppm for the GM method, which is also very close to the experimental value given in Table S9 (135.2 ppm).
